# Mapping past human land use using archaeological data: A new classification for global land use synthesis and data harmonization

**DOI:** 10.1371/journal.pone.0246662

**Published:** 2021-04-14

**Authors:** Kathleen D. Morrison, Emily Hammer, Oliver Boles, Marco Madella, Nicola Whitehouse, Marie-Jose Gaillard, Jennifer Bates, Marc Vander Linden, Stefania Merlo, Alice Yao, Laura Popova, Austin Chad Hill, Ferran Antolin, Andrew Bauer, Stefano Biagetti, Rosie R. Bishop, Phillip Buckland, Pablo Cruz, Dagmar Dreslerová, Gerrit Dusseldorp, Erle Ellis, Dragana Filipovic, Thomas Foster, Matthew J. Hannaford, Sandy P. Harrison, Manjil Hazarika, Hajnalka Herold, Johanna Hilpert, Jed O. Kaplan, Andrea Kay, Kees Klein Goldewijk, Jan Kolář, Elizabeth Kyazike, Julian Laabs, Carla Lancelotti, Paul Lane, Dan Lawrence, Krista Lewis, Umberto Lombardo, Giulio Lucarini, Manuel Arroyo-Kalin, Rob Marchant, Francis Mayle, Meriel McClatchie, Madeleine McLeester, Scott Mooney, Magdalena Moskal-del Hoyo, Vanessa Navarrete, Emmanuel Ndiema, Eduardo Góes Neves, Marek Nowak, Welmoed A. Out, Cameron Petrie, Leanne N. Phelps, Zsolt Pinke, Stéphen Rostain, Thembi Russell, Andrew Sluyter, Amy K. Styring, Eduardo Tamanaha, Evert Thomas, Selvakumar Veerasamy, Lynn Welton, Marco Zanon

**Affiliations:** 1 Department of Anthropology, University of Pennsylvania, Philadelphia, Pennsylvania, United States of America; 2 Department of Near East Languages and Civilizations and the Price Lab for the Digital Humanities, University of Pennsylvania, Philadelphia, Pennsylvania, United States of America; 3 ICREA–CaSEs–Department of Humanities, Universitat Pompeu Fabra, Barcelona, Spain; 4 School of Geography, Archaeology and Environmental Studies, The University of the Witwatersrand, Johannesburg, South Africa; 5 School of Geography, Earth and Environmental Sciences, Plymouth University, Plymouth, United Kingdom; 6 Department of Archaeology, University of Glasgow, Glasgow, United Kingdom; 7 Department of Biology and Environmental Sciences, Linnaeus University, Växjö, Sweden; 8 Institute for the Modelling of Socio-Environmental Transitions, Bournemouth University, Bournemouth, United Kingdom; 9 Department of Anthropology, University of Chicago, Chicago, Illinois, United States of America; 10 Barrett Honors College, Arizona State University, Tempe, Arizona, United States of America; 11 Integrative Prehistory and Archaeological Science (IPNA/IPAS), University of Basel, Basel, Switzerland; 12 Department of Anthropology, Stanford University, Stanford, California, United States of America; 13 Department d’Humanitats, Universitat Pompeu Fabra, Barcelona, Spain; 14 School of Geography, Archaeology and Environmental Studies, University of the Witwatersrand, South Africa; 15 Museum of Archaeology, University of Stavanger, Stavanger, Norway; 16 Department of Historical, Philosophical and religious Studies, Umeå University, Umeå, Sweden; 17 UE CISOR CONICET UNJu, Argentine National Science Council (CONICET), Argentina; 18 Institute of Archaeology of the Czech Academy of Sciences, Academy of Sciences Prague, Czech Republic; 19 Faculty of Archaeology, Leiden University, Leiden, The Netherlands; 20 Palaeo-Research Institute, University of Johannesburg, Johannesburg, South Africa; 21 Department of Geography and Environmental Systems, University of Maryland Baltimore County, Maryland, United States of America; 22 Institute of Pre- and Protohistoric Archaeology, Kiel, Germany; 23 College of Arts & Sciences, Anthropology, University of Tulsa, Tusla, Oklahoma, United States of America; 24 School of Geography, University of Lincoln, Lincoln, United Kingdom; 25 School of Archaeology, Geography and Environmental Science, University of Reading, Reading, United Kingdom; 26 Department of Archaeology, Cotton University, Guwahati, India; 27 Department of Archaeology, University of Exeter, Exeter, United Kingdom; 28 Institute for Prehistoric Archaeology, Universitat zu Koln, Cologne, Germany; 29 Department of Earth Sciences, The University of Hong Kong, Hong Kong, Hong Kong; 30 Max Planck Institute for the Science of Human History, Jena, Germany; 31 Copernicus Institute of Sustainable Development, Utrecht University, Utrecht, The Netherlands; 32 Institute of Botany of the Czech Academy of Sciences, Prague, Czech Republic; 33 Institute of Archaeology and Museology, Masaryk University, Brno, Czech Republic; 34 Department of History and Political Science, Kyambogo University, Kampala, Uganda; 35 Institute for Archaeolgical Scienes, Bern University, Bern, Switzerland; 36 Oeschger Centre for Climate Change Research, Bern University, Bern, Switzerland; 37 Institute of Pre- and Protohistoric Archaeology, Kiel University, Keil, Germany; 38 ICREA–Department of Humanities, Universitat Pompeu Fabra, Barcelona, Spain; 39 Department of Archaeology, University of Cambridge, Cambridge, United Kingdom; 40 School of Geography, Archaeology and Environmental Studies, University of the Witwatersrand, Witwatersrand, South Africa; 41 Department of Archaeology, Durham University, Durham, United Kingdom; 42 Department of Sociology and Anthropology, University of Arkansas at Little Rock, Little Rock, Arkansas, United States of America; 43 Institute of Geography, University of Bern, Bern, Switzerland; 44 Institute of Heritage Science, National Research Council of Italy, Montelibretti, Rome, Italy; 45 Department of Asian, African and Mediterranean Studies, University of Naples L’Orientale, Naples, Italy; 46 Institute of Archaeology, University College London, London, United Kingdom; 47 York Institute for Tropical Ecosystems, Department of Environment and Geography, University of York, York, United Kingdom; 48 Department of Geography and Environmental Science, University of Reading, Reading, United Kingdom; 49 School of Archaeology, University College Dublin, Dublin, Ireland; 50 Department of Anthropology, Dartmouth College, Hanover, New Hampshire, United States of America; 51 School of Biological, Earth and Environmental Sciences, UNSW Sydney, Sydney, Australia; 52 W. Szafer Institute of Botany, Polish Academy of Sciences, Warsaw, Poland; 53 Department of Prehistory, Universitat Autònoma de Barcelona, Bellaterra, Spain; 54 Department of Earth Sciences, National Museums of Kenya, Nairobi, Kenya; 55 Laboratório de Arqueologia dos Trópicos, Museu de Arqueologia e Etnologia, Universidade de São Paulo, São Paulo, Brazil; 56 Institute of Archaeology, Jagiellonian University, Kraków, Poland; 57 Department of Archaeological Science and Conservation, Moesgaard Museum, Højbjerg, Denmark; 58 McDonald Institute for Archaeological Research, University of Cambridge, Cambridge, United Kingdom; 59 Tropical diversity, Royal Botanic Garden Edinburgh, Edinburgh, United Kingdom; 60 School of GeoSciences, University of Edinburgh, Edinburgh, United Kingdom; 61 Department of Physical Geography, Eötvös Loránd University, Budapest, Hungary; 62 Centre national de la recherche scientifique, Nanterre, France; 63 Department of Geography and Anthropology, Louisana State University, Baton Rouge, Louisiana, United States of America; 64 School of Archaeology, University of Oxford, Oxford, United Kingdom; 65 Instituto de Desenvolvimento Sustentável Mamirauá, Amazonas, Brazil; 66 The Alliance of Bioversity International and CIAT, Lima, Peru; 67 Department of Maritime History and Marine Archaeology, Tamil University, Tanjore, India; Utah State University, UNITED STATES

## Abstract

In the 12,000 years preceding the Industrial Revolution, human activities led to significant changes in land cover, plant and animal distributions, surface hydrology, and biochemical cycles. Earth system models suggest that this anthropogenic land cover change influenced regional and global climate. However, the representation of past land use in earth system models is currently oversimplified. As a result, there are large uncertainties in the current understanding of the past and current state of the earth system. In order to improve representation of the variety and scale of impacts that past land use had on the earth system, a global effort is underway to aggregate and synthesize archaeological and historical evidence of land use systems. Here we present a simple, hierarchical classification of land use systems designed to be used with archaeological and historical data at a global scale and a schema of codes that identify land use practices common to a range of systems, both implemented in a geospatial database. The classification scheme and database resulted from an extensive process of consultation with researchers worldwide. Our scheme is designed to deliver consistent, empirically robust data for the improvement of land use models, while simultaneously allowing for a comparative, detailed mapping of land use relevant to the needs of historical scholars. To illustrate the benefits of the classification scheme and methods for mapping historical land use, we apply it to Mesopotamia and Arabia at 6 kya (c. 4000 BCE). The scheme will be used to describe land use by the Past Global Changes (PAGES) LandCover6k working group, an international project comprised of archaeologists, historians, geographers, paleoecologists, and modelers. Beyond this, the scheme has a wide utility for creating a common language between research and policy communities, linking archaeologists with climate modelers, biodiversity conservation workers and initiatives.

*Land transformation has been the primary driving force of human alteration of terrestrial ecosystems*, *strongly interacting with most other aspects of global environmental change*…

                                        *Weindl et al*. *2017*

## Introduction: Earth systems models, land cover, and the past

Although earth system models are often seen as tools for exploring the future, they rely in part on understandings of the past, including models of land cover change through time. It is the aim of the LandCover6k working group, formed in 2015 as a collaboration between archaeologists, historians, geographers, paleoecologists, and modelers, to improve the basis for incorporating past land cover change into earth system models (http://pastglobalchanges.org/science/wg/landcover6k/intro). We seek to do this by producing reconstructions of past vegetation and human land use through time that are grounded in paleoenvironmental and archaeological data.

In order to explain how paleoenvironmental and archaeological data can improve earth system models, it is necessary to review these models and the limitations of their approach to land cover. The land surface is a central component of the earth system. Changes in land cover affect a range of earth system processes including biodiversity, water resources, and air quality. Land cover also influences climate through interactions between land and atmosphere. These may be broadly partitioned into biogeochemical feedbacks, including sources and sinks of greenhouse gases and aerosols, and biogeophysical feedbacks, including surface reflectance (albedo), evapotranspiration, and momentum transfer from wind [1]. Some land-atmosphere feedbacks are positive, amplifying ongoing climate change, while others are negative, attenuating climatic trends. Land-atmosphere interactions currently constitute an area of uncertainty in climate projections, and it is a priority in the scientific community to improve earth system models by incorporating land cover change into simulations.

Understanding land cover change requires information on vegetation and human land use as well as interlinked changes between the two. In earth system models, this information comes from dynamic vegetation schemes and anthropogenic land cover change scenarios. Recent earth system models contain a dynamic vegetation scheme that simulates the distribution and properties of potential natural vegetation, i.e., vegetation that would be predicted to grow under specific climatic and soil conditions [2, 3]. These models allow land cover to change with climatic change and simulate how land cover and climate interact through positive or negative feedbacks. Dynamic vegetation schemes do not necessarily reflect actual land cover, because human agency often leads to modification of land cover that cannot be predicted on the basis of environmental change alone. Scenarios used to make future climate projections therefore employ a *representation* of anthropogenic land cover change, including deforestation and expansion and abandonment of cropland, as an essential boundary condition [4–8]. While there are numerous proxies for paleoenvironmental change, their distribution in space and time is highly heterogenous and at present the only way to produce a spatially and temporally continuous picture of past environmental change is to use models. While changes in natural vegetation cover can be simulated directly by many earth system models, as noted above, information on past land use in these models is currently provided by what are called anthropogenic land cover change (ALCC) scenarios [9–13]. The models behind these scenarios generally combine estimates of historic human population figures with model-specific algorithms, based on land use per capita figures, to estimate the total magnitude and spatial distribution of land use. The result of most ALCC models are maps (scenarios) quantifying a general metric of land use such as crop or pasture area fraction.

While ALCC scenarios have been widely applied in earth system modeling studies, all existing ALCC models are subject to major limitations. Most ALCC models are not directly based on proxy data for past land cover, they do not use observations of past vegetation, and they do not incorporate evidence for the variable impacts of past populations on their environments, variability tied to specific land use practices and to social and political factors such as past surplus production, capital accumulation, and trade [2,cf. 14]. Instead, these models presume a parameterized representation of per capita land use that may be constant, time dependent, or dependent on other factors such as population density. All current ALCC scenarios lack thematic information on the effects of past human land use, such as those created by large-scale burning, plowing, irrigation, and livestock management. Finally, ALCC scenarios differ significantly from one another and compare poorly with independent reconstructions [15]. In spite of these inherent limitations, ALCC models are used as part of Land Use Harmonization Models [10]. It is therefore of crucial importance that ALCC models are improved, as ALCC scenarios provide data essential for earth system modeling of the past, present, and future.

A major current research challenge crosscutting the social, biological, and physical sciences is thus to improve our understanding of the scope of early human land use, resultant changes in land cover, and consequent feedbacks to both cultural and climatic systems. Archaeologists and environmental historians have generated a large amount of data on past human land use; they therefore have a central role to play in the improvement of ALCC scenarios. By bringing together this significant repository of archaeological and historical data on how human activities have affected the earth system, archaeologists and historians can contribute to the development of ALCC models that better reflect the timing, magnitude, and nature of human influence on the earth system over time.

The exclusion of archaeological and historical data from ALCC models is not an oversight. Archaeologists and historians have rarely attempted to generate global data in a format that would be useful for a comparative world history of land-use systems or for incorporation into the models of the earth systems science community. Generating such data is a difficult task, requiring the synthesis of heterogeneous qualitative and quantitative datasets into general regional narratives of historical land use through time and then translating such narratives into spatial form within a digital map and database. Additionally, such work should aggregate data on the effects of past land use systems, including changes in land cover and perturbations to biogeochemical cycling through water management, changes in species composition, and disturbance such as fire. Taking on this enormous task, however, is essential both to improve ALCC models and to empower archaeologists and historians to play a larger role in the writing of critical narratives of human-environmental interaction.

In this paper, we lay the groundwork for archaeological and historical synthesis of data on past land use by defining a land use classification scheme. The land use classification is the outcome of more than three years of consultation with groups of archaeologists, historians, and geographers from all parts of the world who specialize in time periods covering the entire Holocene. While a preliminary version of our classification was presented by Morrison et al. [2], here we provide more detail on the classification, its application, its implementation in a geospatial database, and the research processes required to start a global mapping effort.

### Holocene land use and its significance: LandCover6k

The degree to which pre-industrial land use and consequent land cover change affected the global climate is disputed [16–20]. Some human-induced changes were dramatic, such as large-scale forest clearance and management; the domestication of plant and animal species, the establishment of associated agricultural livelihoods, and the redistribution of these across the planet; and the reshaping of entire environments via terracing, irrigation, and urban expansion. Other transformations such as the management of wild plants, hunting, and the long-term use of fire on a regional scale [21] are less evidently consequential but may, in aggregate, have also contributed to earth system-level changes. These pre-industrial anthropogenic influences on land cover and biogeochemical cycling may have affected climate through both biogeophysical and biogeochemical feedbacks [22].

There is little doubt that the effects of pre-industrial human land use on terrestrial ecosystems were profound at local to regional scales, e.g., with the advent of agricultural societies in Southwest Asia, South Asia, China, and Mesoamerica [for summary of discussions, see 23], but global scale effects are more debated. Several existing ALCC models suggest that vegetation modification was fairly minor at global scales prior to the Industrial Revolution [9, 22, 24], with correspondingly small greenhouse gas releases and minimal effects on the global carbon cycle and climate system [25]. Others have argued that early agricultural and pastoral activities triggered significant releases of greenhouse gases (CO_2_, CH_4_) to the atmosphere [e.g., 11, 26, 27]. Kaplan et al. [11] estimated that Holocene ALCC could have resulted in 84 to 102 Pg C released to the atmosphere by 3000 BP, equivalent to a substantial rise in atmospheric CO_2_ of 7 ppm. These estimates are consistent with measurements of the isotopic composition of CO_2_ recovered from high-resolution Antarctic ice-cores [25] because for much of the Holocene anthropogenic CO_2_ emissions were offset by the long-term sequestration of carbon due to peatland expansion [35].

In many places, the intensity of land use practices increased over time through the Holocene [e.g., 28, 29], although this process was by no means uniform across time and space. Land use choices and their consequences, such as effects on vegetation, soils, and wildlife, are path-dependent, meaning that they are contingent upon complex and recursive sets of prior conditions, including past human land use itself. While extreme environments historically limited land use options, those limits also fluctuated as a result of technological change and past climate change. Thus, land use at any one time is influenced by land cover, precipitation, soil fertility, and other ‘natural’ situations that create suitable conditions, but technological developments can potentially transform those conditions. Over the 12,000 years of the pre-industrial Holocene, human populations expanded their distribution to permanently settle on all of the continents except Antarctica, and on most oceanic islands. While increasing population did play an important role in the expansion of human impact, numbers alone are misleading, as factors such as wealth accumulation, inequality of consumption, and cultural demands for specific goods and produce also fueled significant land use changes [30]. Anthropogenic effects on land cover therefore cannot be reduced to population variables alone; instead, levels and forms of consumption have also played a significant role. That is, not all people have, or had, identical impacts on the environment [31]. Differential forms and rates of consumption are made possible by land use practices (e.g., farming) and by other forms of production (e.g., metal working). Land use, the mechanism by which resources such as food, fuel, and other goods (e.g., prestige items, technological items) are produced, emerges as a critical mediator between ‘raw’ population human numbers and realized environmental impacts [32].

A great deal of uncertainty still surrounds the Holocene CO_2_ record, and this uncertainty is fostered by the lack of high-quality data-based syntheses of global land use and of anthropogenic land cover change for the Holocene. Syntheses of historical land use are less developed than those of land cover, in part because of higher data heterogeneity in the archaeological and historical record and the larger size and disciplinary diversity of the scholarly communities involved. While numerous regional-scale syntheses exist [e.g. 33, 34], even world prehistory or global history textbooks generally do not attempt to integrate land use information using a consistent format for all periods and regions. Significant efforts to aggregate and synthesize archaeological and historical information in publicly available databases exist for North America (DINAA–Digital Index of North American Archaeology; CARD–Canadian Archaeological Radiocarbon Database) and Europe (e.g., The Cultural Evolution of Neolithic Europe EUROEVOL, [35]) but these are typically limited in spatial and temporal scope. These important databases of site-level records are not available for all world regions. A recent effort at global-scale synthesis from archaeological data [29] using a preliminary version [2] of the classification presented here established the value of such efforts, but did not produce a fine-grained, empirically consistent data set, nor did it attempt to integrate historical and archaeological evidence of past land use with information from other proxies such as pollen records [36].

These lacunae are addressed through the LandCover6k project, which is working to produce global maps of land use and land cover based on synthesized archaeological and paleoecological data pertaining to designated time slices throughout the Holocene, from the advent of farming to the industrial revolution [37]. Mapping both human land use and land cover allows us to reconstruct anthropogenic land cover change (ALCC) directly, using the rich empirical records of both paleoecology and archaeology. LandCover6k consists of three interrelated efforts: (1) *land cover synthesis and mapping* [e.g., 38], using pollen records and pollen-vegetation models such as REVEALS [39] and others [40]; (2) *land use synthesis and mapping* [2 and this paper]; and (3) *modeler-paleoscientist coordination and co-design* [8, see [41] for a general discussion of the LandCover6k project]. Empirically independent records of land use and land cover are needed in order to better understand the complex relationships between them. If we are to resolve the debate over the longer-term impact of humans on the earth system, the forms, timing, extent, severity, and significance of human action on land cover and other processes such as carbon cycling cannot be assumed; they must be empirically demonstrated. This paper outlines our approach to land use synthesis and mapping, designed specifically to be integrated with the work of the land use and earth system modeling efforts that are part of LandCover6k [8] and to integrate with pollen-based land cover syntheses [see [42] for an example set in Ireland].

## Classifying past land use

The goals of the LandCover6k project required the development of a bespoke land use classification scheme. Many existing classifications of anthropogenic land use focus more on the outcomes or presumed outcomes of these activities than on the activities themselves. Sauer, for example, defined five land utilization categories [43 pp. 48]: “(1) barrens, (2) woodlands, (3) permanent pastures and meadows, (4) cultivated lands, [and] (5) town sites.” While Sauer’s categories have found broad application, e.g., in the concept of “anthromes”, the specific goals of the LandCover6k project required a purpose-built classification that separates cultural activities from possible outcomes and that specifies human land use practices more explicitly. This is required because more than one form of land use can result in the same land cover. For example, a woodland could be created through land use practices as different as foraging and arboriculture. Because a fundamental goal of LandCover6k is to better understand land use and land cover changes and their interconnections [37], we developed a land use classification focused on cultural practices, i.e., the ‘uses’ people made of the land, rather than on the presumed outcomes of those practices [cf. 44].

Although related, land use is distinct from land cover [45], but many existing land use classifications do not fully separate the two [46, 47]. The conflation of the two concepts creates both ontological and epistemological issues with consequences for the classification and its usability [45]. Data from satellite and aerial imagery, for example, provide information on the biophysical properties of a land unit from which land use is then inferred. Classification structures derived from remote sensing often employ terminology applicable to both land use and land cover, such as ‘cropland’ or ‘forest’ [e.g. 10, 48]. However, forests are a land cover category which can be used in many ways such as recreation, hunting, foraging, or forms of agroforestry. Thus, to understand how past land use affected forests, we need independent evidence for both the land use (in the form of archaeological or historical data) and the land cover (from paleoecological data such as pollen analysis).

Ethnographic descriptions of traditional land use systems are an important basis for understanding past land use, but they are not sufficient. There are past land use systems that lack modern or ethnographic analogues. Further, the global scope of LandCover6k requires collapsing regionally and temporally specific vocabulary into fewer and more generic categories. For example, the category of swidden/shifting cultivation is variously known as slash-and-burn, *ladang*, *milpa*, *jhum* and other terms [49]. These terms have only local salience and are not used with any consistency on a larger spatial scale. There is variability within swidden/shifting forms of cultivation with respect to cultigens, the period of active cultivation and of fallow, and the extent to which woody plants are integral to cropping regimes, but these land use systems are similar enough to be united for large-scale classification of land use effects on land cover. Although our classification uses terms current in the literature, we have favored generic rather than temporally or regionally specific terms, and a major effort has gone into defining these.

Our first task was the development of a uniform terminology for a single land use classification that could be used for all time periods and regions. Historical and archaeological data on past land use are extensive, but widely scattered, based on a diverse range of indicators, and interpreted using multiple classification systems. Definitions of land use categories change from context to context, and between different archaeological and historical traditions. These challenges may make scholars reluctant to commit to definitive land use categories. However, the detailed and nuanced understandings of past land use developed for individual regions must be simplified in some way to be used for global-scale description of land use changes. Our classification is designed to build connections between the terms, procedures, and forms of knowledge produced by highly diverse scholarly communities. Using archaeological and historical data in ALCC models requires harmonization of land use categories, but existing ALCC schema [9–13, 50, 51] use a terminology that was not designed to incorporate archaeological data. Bridging the gap between the data-rich but classification-diverse world of historical scholarship and the ALCC community is important if the latter are to benefit from the long history of scholarship on past land use.

Our classification of agricultural types shares some features with the work of Widgren and colleagues [52, 53; see also 54, 55] who produced global maps of the dominant agricultural systems between CE 1000 and 1800, but it also differs in significant ways. Widgren and colleagues used a limited number of categories since the aim was to produce global maps of dominant land use systems. LandCover6k instead produces a database, which means that it can include more information than might be legible on a map. We also opted to analytically separate systems of production into components. For example, the co-occurrence of domesticated plants and animals in an agrarian system is often defined as ‘mixed farming’ [52], but there is little agreement on how much emphasis on livestock (and of what kind) is needed to qualify a system as mixed. Numerical thresholds (e.g. “50% of the income produced on farm should come from livestock”) are not something archaeologists can calculate with any degree of certainty [though see 56–58]. We therefore code livestock-keeping separately from the cultivation of domestic plants in the database, with the understanding that these often co-occur. Our data structure allows us to track these correlations without needing to define them *a priori*.

We also built on prior classifications of hunting and gathering and pastoralism [59–61] but in a way that is adapted to the possibilities and limitations of archaeological evidence and accommodates a database structure that includes numerous additional variables. Khasanov’s [62] categorization of pastoralism, for example, is a useful framework to break down livestock management practices according to levels of mobility; spatial and temporal patterns of movement between resource zones are clearly a significant factor in the potential land cover impact of pastoralism. However, archaeological data cannot necessarily identify levels of mobility in the past [63]; where this is possible, it requires datasets that scholars have only just begun to collect [64, 65].

Past land use is inferred from multiple forms of archaeological and historical data [66]. Pastoral practices with or without concomitant forms of agriculture, for example, may be identifiable on the basis of settlement sizes, distributions, and duration [65, 67, 68]; faunal remains [showing exploitation of wild taxa, husbandry practices of domesticates, including the use of secondary products such as milk, traction, and manure; e.g., 69–72]; botanical remains [macro- and micro-remains showing crops grown, commensal weeds, sowing, cultivation, processing and storing strategies, taxonomic signatures of wood assemblages from fuel; 73–77]; coprophilous fungal spores, dung and pasture/meadow fossil beetles and dung residues indicative of large grazers [e.g., 78–82]; landscape features [relict fields, terraces, canals, reservoirs, check-dams, 83–88]; geoarchaeological evidence [soil micromorphology, buried soil profiles, evidence of erosional regimes; e.g., 89–92]; and isotopic evidence for human and animal diet and cultivation practices [e.g., 64, 75, 93–96]. Archaeological data are unevenly distributed in space and time and subject to different taphonomic pressures. Building on various typologies previously assembled under the auspices of LandCover6k [e.g., 2, 97–99], the system presented here harmonizes and synthesizes the literature on human subsistence practices and other land use activities.

### The LandCover6k land use classification and variables

The LandCover6k land use classification scheme has five principal features. First, it is scale- and source-independent. Second, it uses uniform terminology for all world regions and periods, terminology that was agreed upon in LandCover6k workshops involving diverse participants. Third, it is hierarchical and flexible, incorporating both categories of varying specificity and variables that are relevant across land use systems. The categories and variables are defined with the limitations of archaeological data in mind, specifically the variability in data coverage and data quality across regions and time periods. Variables also include assessments of data coverage and quality. Fourth, it relies upon expert assessment of dominant forms of land use in an 8 x 8 km area at a particular point in time (see “*Implementation of the Classification in a Geospatial Database*,*” below)*. Fifth, the classification takes the perspective of land rather than people. We explain and justify each of these features below.

According to the Food and Agriculture Organization of the United Nations (FAO), land cover classifications should be “scale-independent, meaning that the classes at all levels of the system should be applicable at any scale or level of detail; and source-independent, implying that it is independent of the means used to collect information” [100]. Following these principles, our database classifies global land use according to predefined categories at varying levels of detail. Within the hierarchical structure of the classification (discussed further below), all categories are scale-independent and independent of the different data sources used. Source-independence is especially important for classifications of human land use practices since there are so many ways to study them, as described above. Because not all forms of information are available or relevant for all times and places, it is not feasible to build categories *a posteriori* by recording huge amounts of primary data. Instead, our categories are *a priori*, using terms widespread in the literature, modified and refined by an extensive process of consultation and workshops, and harmonized into a single globally applicable system.

The classification employs consistent language for describing historical and ancient land use. As discussed previously, a frequent obstacle to the global synthesis of past data on land use has been historians’ and archaeologists’ tendency to use terminology specific to their region and time period of interest. This is further complicated by the wide range of disciplinary traditions concerned with the collection of land use data and the diverse forms of information each can contribute. We have attempted to adopt language that preserves some of the complexity and nuance contained within existing terminology but also simplifies enough to facilitate global comparisons.

The structure of the classification is hierarchical. Categories are divided into levels termed ‘LU’ (land use) levels 1–3. The highest level of classification, LU1, is the most general and is designed to facilitate broad global analyses, while second- and third-order categories, LU2 and LU3, provide the opportunity to record increasingly detailed information suitable for more nuanced studies. The LU1 classification applied to a grid cell is the dominant type of land use at that grid cell location and is the most relevant for modelling purposes. The more specific levels of classification (and the potential expandability of the classification at LU3), allow scholars to pursue regionally or temporally specific analyses but also have their data included in global-scale studies.

While we do not explicitly account for the intensity of land use activity, in areas where human beings were present to only a limited extent, for example only through transit routes or access to restricted areas, we propose a classification of ‘extensive or minimal land use’. While this category implies minimal impact of human activity, it is important to distinguish such zones from areas with ‘no human land use’, such as unpopulated islands and high-altitude zones.

A challenge of historical land use mapping is that the boundaries between or among land use classes can be ambiguous. There is, for example, a robust debate in the archaeological literature about the definition of agriculture, with many scholars suggesting that food procurement and food production be viewed as a continuum [101–103]. We cover this middle ground with ‘Low Level Food Production,’ a LU2 category that can be used to highlight times and places where land use practices straddled the divide between these subsistence strategies, recognizing that neither is necessarily exclusive [103].

Further to our hierarchical land use classification, we define additional variables that are relevant across land use categories. These variables are vital to understanding the relationship between land use and land cover and are designed to assist climate modelers in addressing specific concerns such as the history of landscape burning, livestock, soil turnover, wood harvest, and other issues. Again, extensive consultation between archaeologists and climate modelers was necessary to develop categories and measures that are both useful for earth systems scientists but also amenable to archaeological research. As noted, recording variables as well as categories allows us to make some land use categories more generic. For example, the term ‘agropastoralism’ is widely used to describe land use systems that integrate both farming and animal husbandry, especially those with ‘high’ levels of dependence on domesticates such as ovicaprids and/or bovids. However, the term is not used consistently around the world and there is no agreed-upon standard for what balance of farming and herding constitutes ‘agropastoralism’ and what is simply ‘agriculture’ with some domestic animals. Accordingly, we coded for animal (and plant) domesticates as variables distinct from the classification of agricultural practices, allowing for a higher degree of variability to be recorded. The variables recorded across land use systems include assessments of data coverage and quality in order to facilitate identification of times and places needing additional research.

Human land use is often heterogeneous, integrating multiple forms and strategies of production [49]; our scheme is structured to capture some of that diversity, but of necessity requires that decisions about dominant land use practices be made, sacrificing detail for scale, especially in the higher levels of classification. Our use of a relatively fine-grained 8 x 8 km spatial grid (discussed below in “*Implementation of the Classification in a Geospatial Database”*) is also meant to allow land use mosaics to be more faithfully represented than they are at coarser spatial resolutions. In some cases, archaeological and historical data are capable of making even finer spatial distinctions than can be captured by the grid, but in other cases the data are not yet adequate to this task. Our analytical practices are thus a compromise–without sacrificing the primary goal of producing empirically-grounded land use maps for climate modelers, we also worked to develop recording strategies that would be of value to archaeologists, historians, human geographers, environmentalists, and others.

It is important to stress that this land use classification pertains specifically to *land rather than people*. We are mapping the forms of past human land use that took place in specific locations and times based on assessments drawing from multiple lines of evidence, as noted above. The specific cultural identity or identities of people in the past who practiced some form of land use is not at issue here. For example, our ‘pastoralism’ category is restricted to areas where very little or no land is under cultivation. Where crops *and* livestock are present, whether or not these are managed by single or multiple social groups, classification falls under the agriculture category, with a specification of which domestic animals are present, as noted above. This “land, not people” distinction is important since most archaeological data is collected and analyzed with reference to cultural or political groupings, and scholars work hard to differentiate the presence of multiple cultural groups in the same place at the same time. Our database does not take these distinctions into account, focusing instead on the aggregate effects of land use practices in one time and place.

Although the focus of the classification is necessarily on land rather than people, we recognize that all types of societies up to the present engage in some use of wild resources, often alongside the use of agricultural and/or pastoral resources, and have built this fact into the definition of our land use categories. Our hunting-gathering-foraging-fishing (HGFF) class only applies if it is the dominant form of land use in a particular area. We preserve this category for land inhabited by people for whom wild resources were/are their principal economic resource [104, 105]. No global map exists of specialized HGFF land use in the past. By adding proxies into our database, such as settlement mode and fire, we can better understand the role of HGFF land use on land cover throughout time. We wish to make the important point that HGFF societies have impacted the landscapes they inhabit–sometimes considerably so [e.g., 106], and we aim to better assess the nature of these impacts. For example, we use the ‘fire: landscape-burning’ variable to signify land management using fire, which was one of the most widespread methods of deliberate land management used by HGFF societies in the past and present [104, 107].

**Fig 1:**
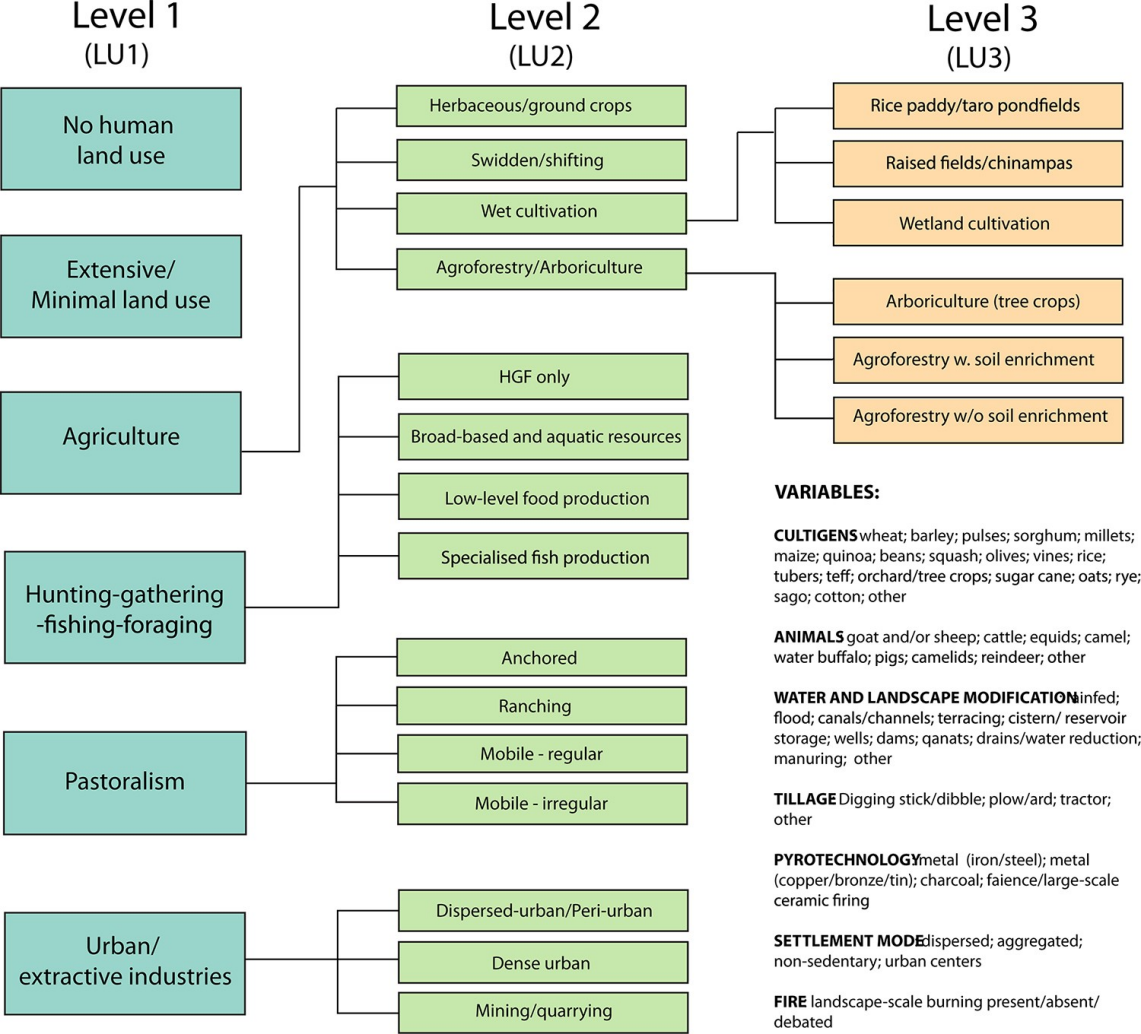
The PAGES LandCover6k land use classification system.

**Fig 2:**
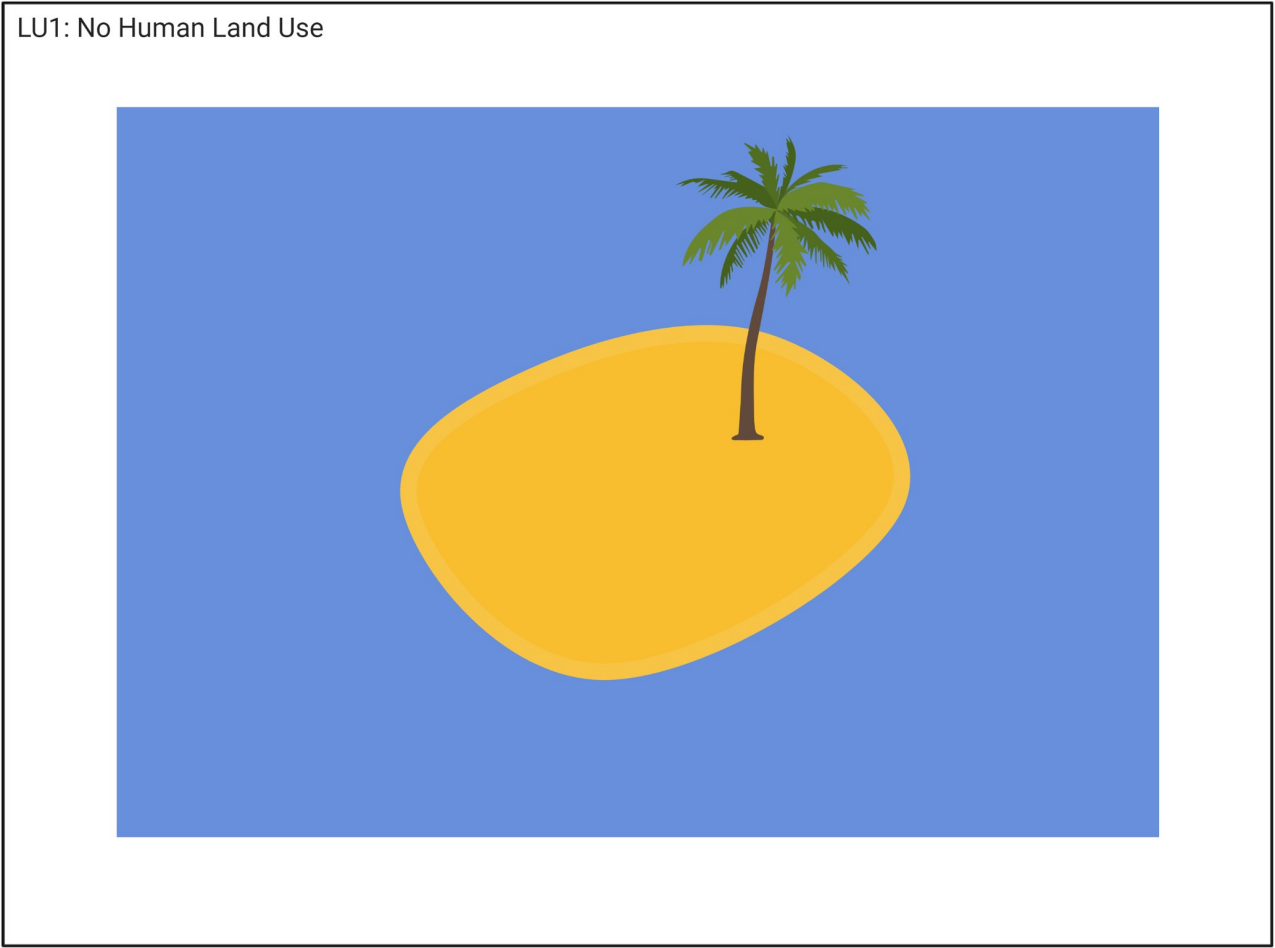
Uninhabited island representing LU1-“no human land use”. Created with BioRender.com, under a CC BY license, with permission from Biorender, original copyright 2020.

**Fig 3:**
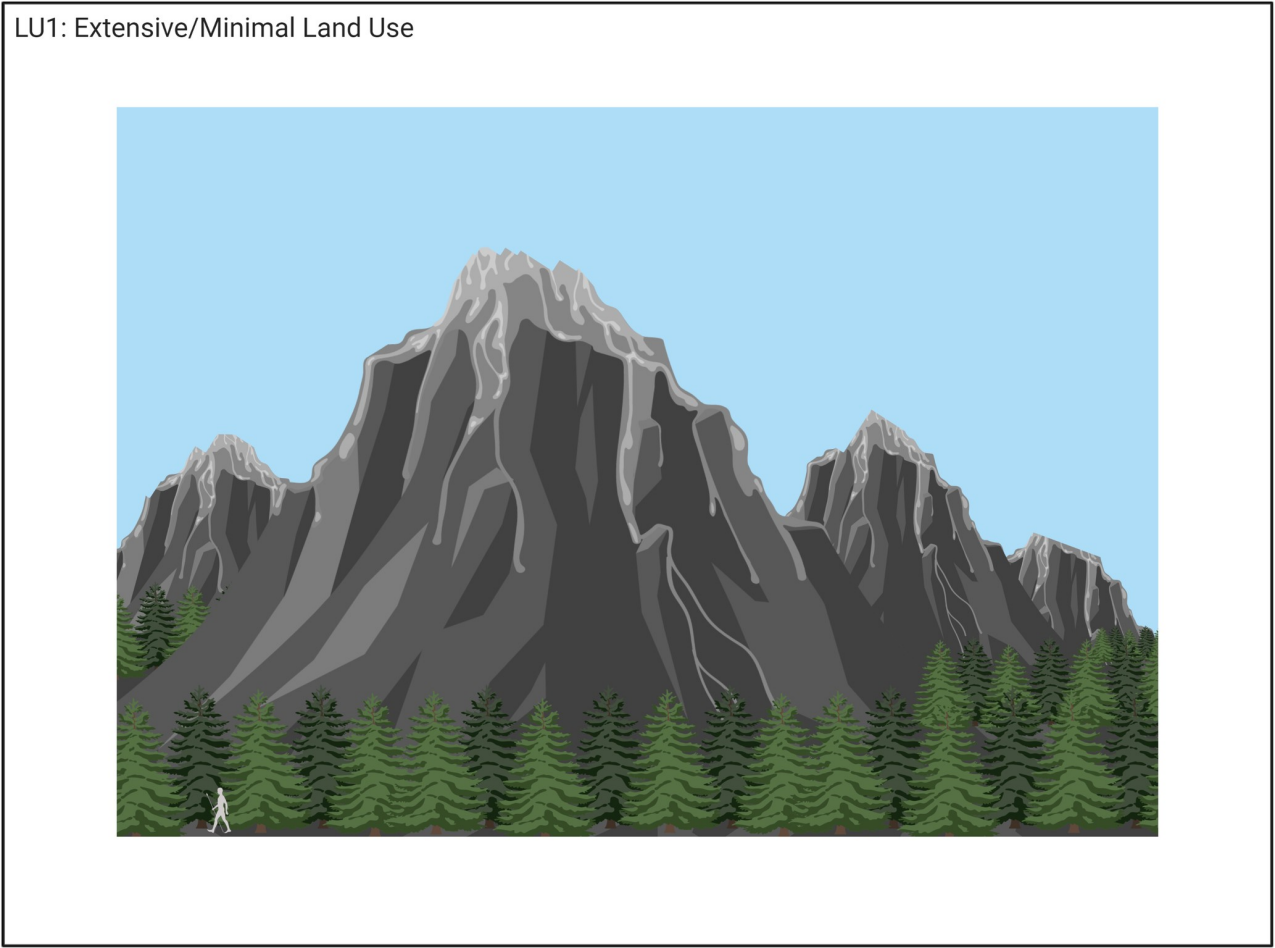
Mountain ranges with access routes or evidence of exploration but not inhabitation representing LU1-“extensive-minimal”. Created with BioRender.com, under a CC BY license, with permission from Biorender, original copyright 2020.

**Fig 4:**
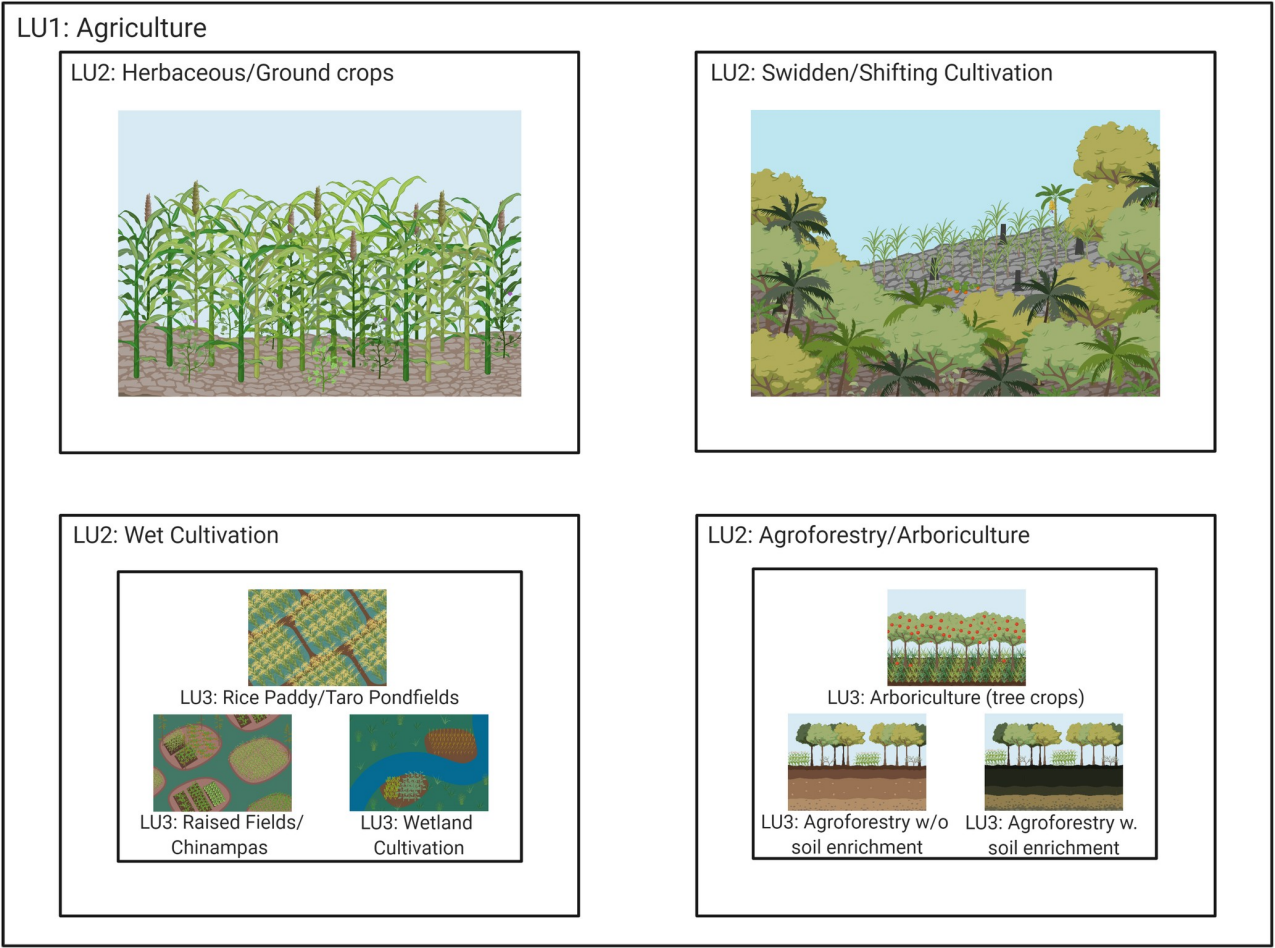
LU1-“agriculture” shown as several LU2 categories: LU2-“herbaceous/ground crops” is shown by agriculture of maize and millets (upper left). LU2-“swidden/shifting cultivation” is shown by forest clearance and cultivation of small crops in that space (upper right). LU2- “wet cultivation” (lower left) includes LU3 categories: LU3-“rice paddy/taro pond fields” represented by rice paddies, LU3-“raised fields/chinampas” shown by chinampa agriculture and LU3-“wetland cultivation” shown by cereal and pulse cultivation on river floodplains. LU2-“agroforestry/arboriculture” (lower right) includes LU3 categories: LU3-“arboriculture (tree crops)” shown with an apple orchard, LU3-“agroforestry” without soil enrichment shown through a woody perennial management system with undifferentiated soils from unexploited areas, and LU3-”agroforestry with soil enrichment of woody perennial management” with a Amazonian dark earth soil. Created with BioRender.com, under a CC BY license, with permission from Biorender, original copyright 2020.

**Fig 5:**
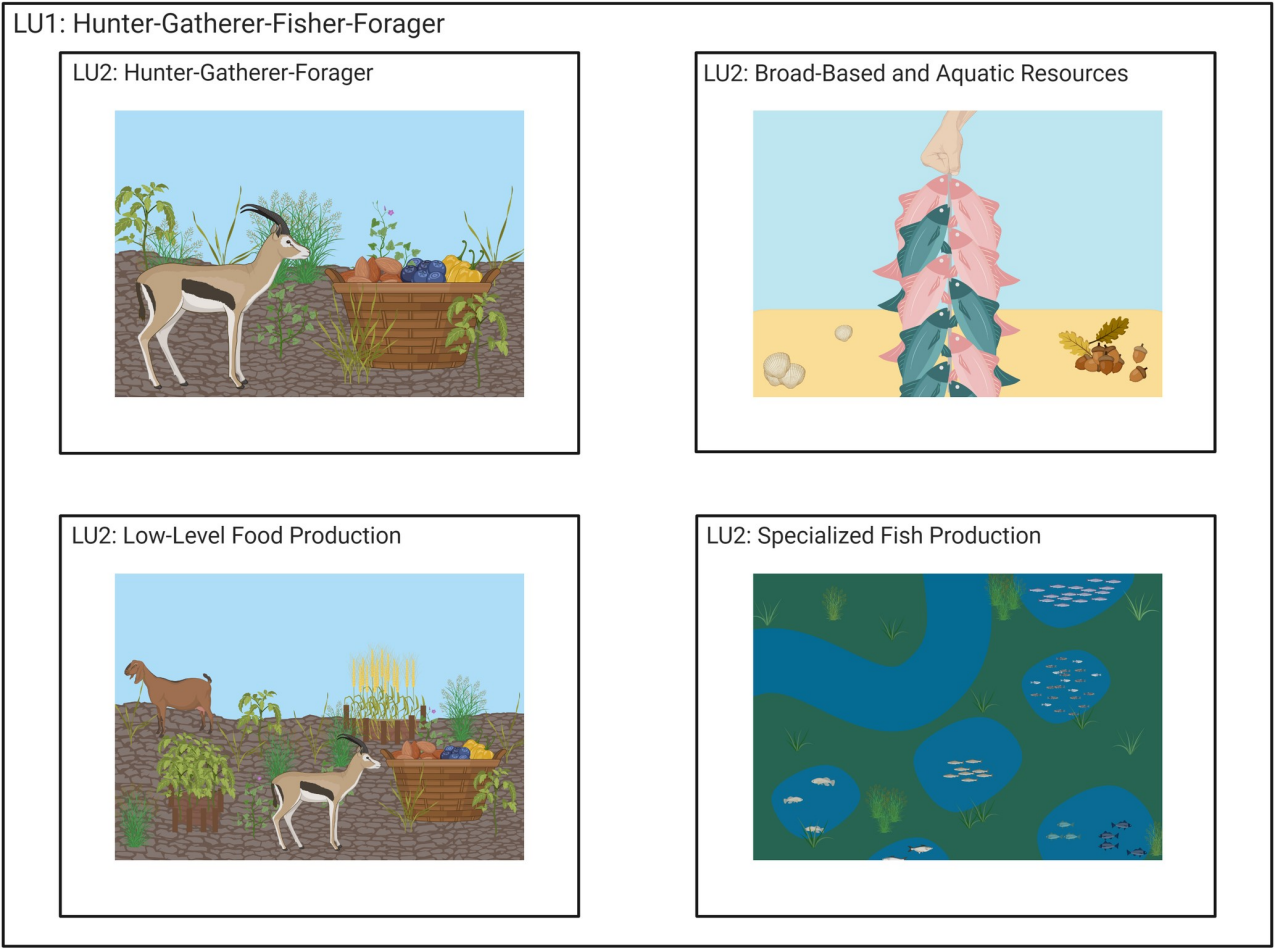
LU1-“hunter-gatherer-fisher-forager” visualized as several LU2 categories: LU2-“hunter-gatherer-forager” is represented by wild resources gazelle and wild gathered nuts, fruits, seeds and berries. LU2-“broad-based and aquatic resources” is shown by a line of caught fish, shells and a collection of gathered nuts. LU2-“low-level food production” is represented by gazelle and wild gathered nuts, fruits, seeds and berries, and a small number of domesticated resources including deer and weedy/semi-domesticated pulses and cereals. LU2-“specialized fish production” is shown through ponded resources in which fish have been collected and placed for future use. Created with BioRender.com, under a CC BY license, with permission from Biorender, original copyright 2020.

**Fig 6:**
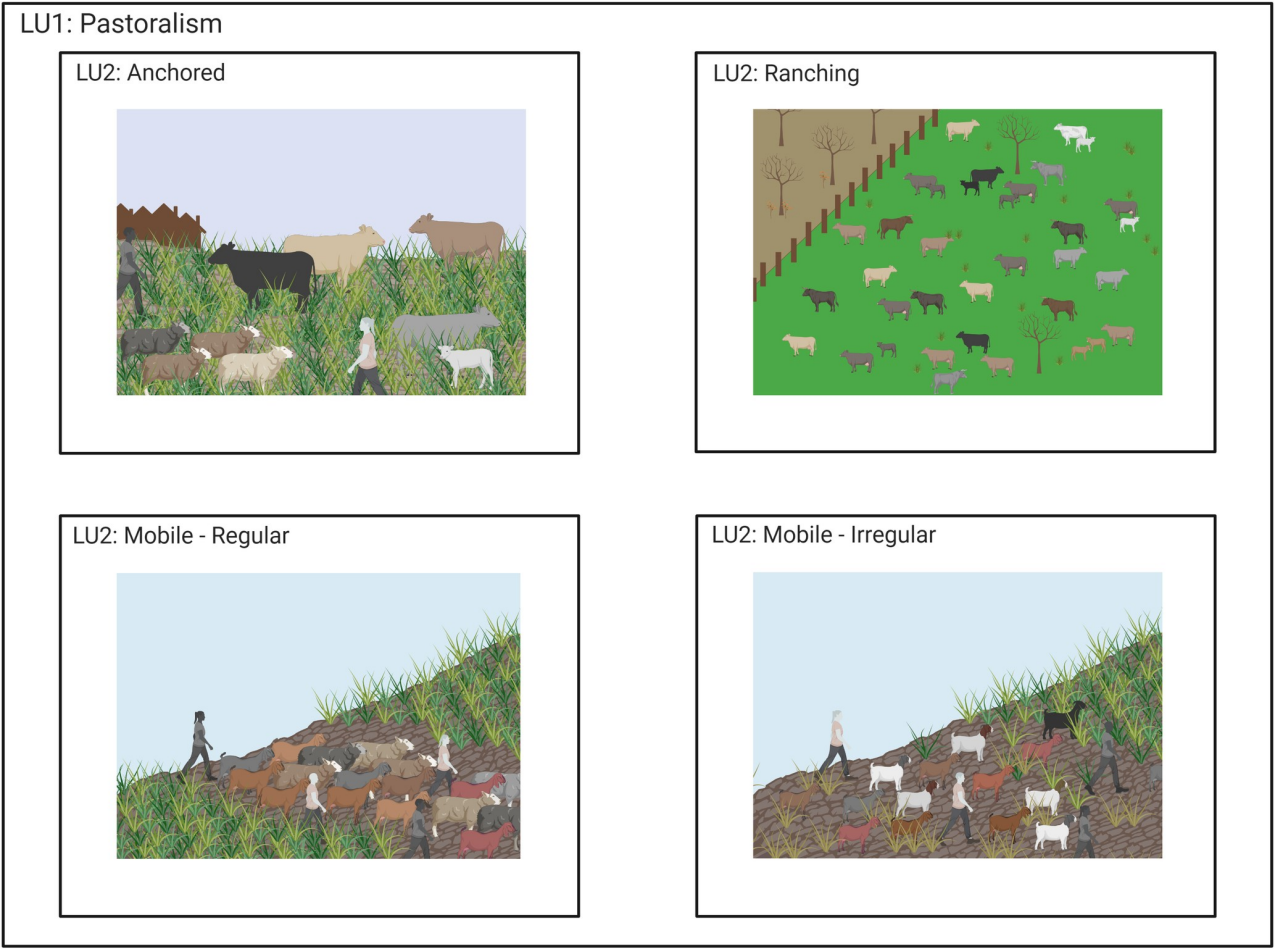
LU1-“pastoralism” shown as several LU2 categories: LU2-“anchored pastoralism” shown as cattle and sheep in proximity to a settlement. LU2-“ranching” shows cattle enclosed in pasture land away from wild/unmanaged lands. LU2-“mobile–regular” shows sheep and goats being led along a specific path. LU2-“mobile–irregular” shows sheep and goat being moved along in a less regular pattern along a less well trodden path. Created with BioRender.com, under a CC BY license, with permission from Biorender, original copyright 2020.

**Fig 7:**
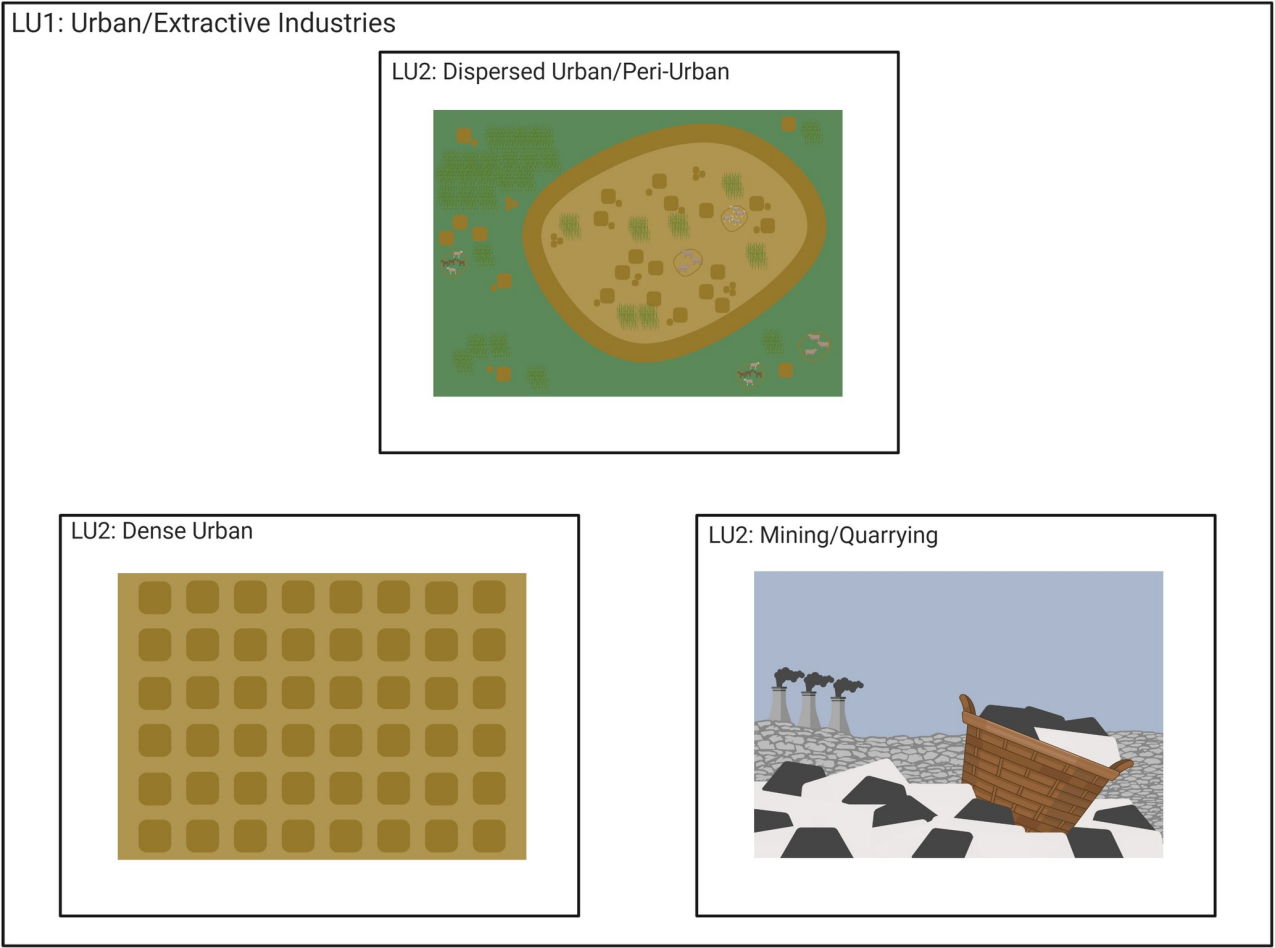
LU1-“Urban/Extractive industries” shown as several LU2 categories: LU2-“dispersed urban/peri-urban” shows a spread-out settlement with houses beyond the wall/edge of the settlement, and agriculture space within the settlement limits. LU2-“dense urban” shows closely packed houses/buildings with little green/agricultural spaces. LU2-“mining/quarrying” is represented by a stone mine. Created with BioRender.com, under a CC BY license, with permission from Biorender, original copyright 2020.

In (S2 File), we describe each upper level (LU1) classification and then, for those categories with LU2 and LU3 distinctions, we explain how those distinctions were made and provide specific examples of that category or subcategory. Listed examples are illustrative rather than exhaustive. Fig 1 shows the land use classification as a hierarchy, with coded variables in list form. Another way to visualize the classification is as a nested series of categories. Schematic figures of each LU1 and its nested subcategories, if any, are included here in the main text to provide illustrations of some of the basic distinctions between them [Figs 2–7].

The classification system has been through various iterations, and the version presented here is the full, final version of the concept note previously published by our working group [2]. While the rationale behind the categories remains the same and we hope to have preserved the allowances for regional examples expressed by the LandCover6k membership, the current system has been modified in accordance with issues raised during testing of the database itself.

## Implementation of the classification in a geospatial database

The LandCover6k project is committed to producing a global land use database for several time slices requested by the climate modelers in joint meetings: 12 kya, 6 kya, 4 kya, and CE 1500. Some continental-scale subgroups are also working on additional time slices. Towards this goal, we have implemented the global land use classification scheme described above and in the (S2 File) in a GIS database, the structure of which is discussed in SI-2. The classification system has been set in the geodatabase in the form of drop-down menus containing all of the valid options for each level of the land use classification hierarchy (LU1, LU2, LU3) and all of the valid options for each of the variables and data assessments relevant across land-use systems.

**Fig 8:**
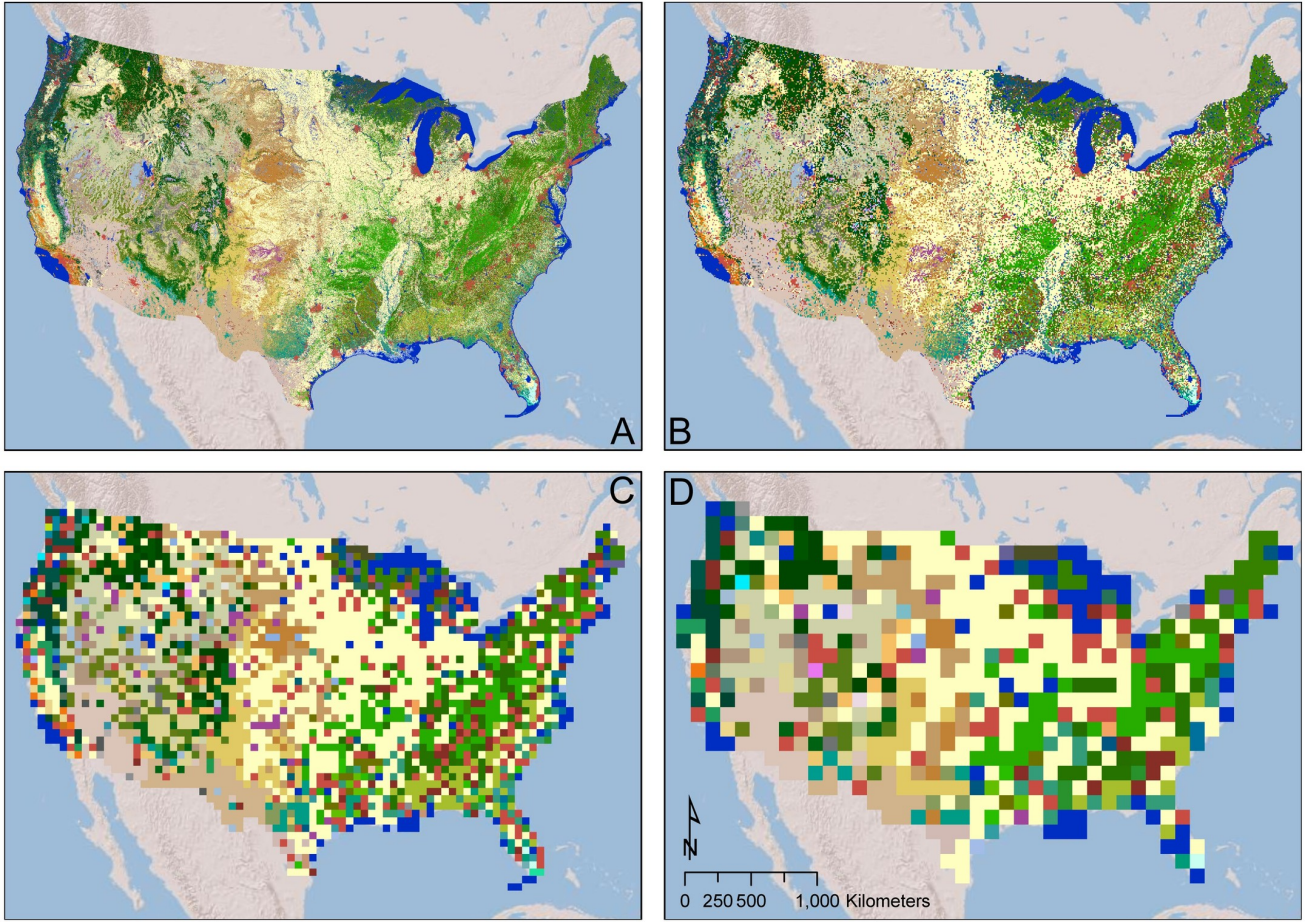
The effect of grid size on data visibility. Modern 30 x 30 m landcover data from the GAP/LANDFIRE National Terrestrial Ecosystems data set (courtesy of the U.S. Geological Survey - https://doi.org/10.5066/F7ZS2TM0) (A), aggregated via majority rule to 8 x 8 km (B), .5° x .5° (C), and 1° x 1° (D) grids.

In order to ensure global coverage and maximum standardization of our land-use maps, regional groups will enter data for 8 x 8 km squares in a vector data format (polygon GIS feature class). The size of spatial units represents a compromise between the needs of the modeling, pollen, and archaeological communities involved in the LandCover6K project. While the LandCover6k pollen group is producing land cover rasters with a resolution of 1 degree [e.g. 108], we elected to use a much smaller grid. The 8 x 8 km grid squares are already much larger than archaeologists and historians are typically comfortable with (as they often spend their whole careers studying individual locales), but we had to compromise in the interest of achieving global maps in a reasonable time frame. The smallest grids used by the ALCC modelers are 5 minutes (1/12 of a degree) so we chose a grid that closely matches this (Fig 8). The task of the regional land use groups will be to enter classifications and to code variables for land that falls within the existing polygon squares.

Each regional subgroup within LandCover6k works with an identical copy of the database to record assessments for squares and time periods falling within the range of their collective expertise. A blank version of the geodatabase with the 8 x 8 km global grid is included in the supplementary material. Because the nature and quality of archaeological data vary by region, different groups develop and pursue their own intermediate strategies of land-use mapping to populate the LandCover6k database. For example, some regional groups are able to rely on pre-existing site databases, while others must first generate them or perform land-use assessments in a more general way. Some groups pursue a strategy of having collaborators draw land use polygons on paper maps or in Google Earth before using the spatial intersection between these polygons and the squares of the global grid to insert classifications into the database.

**Fig 9:**
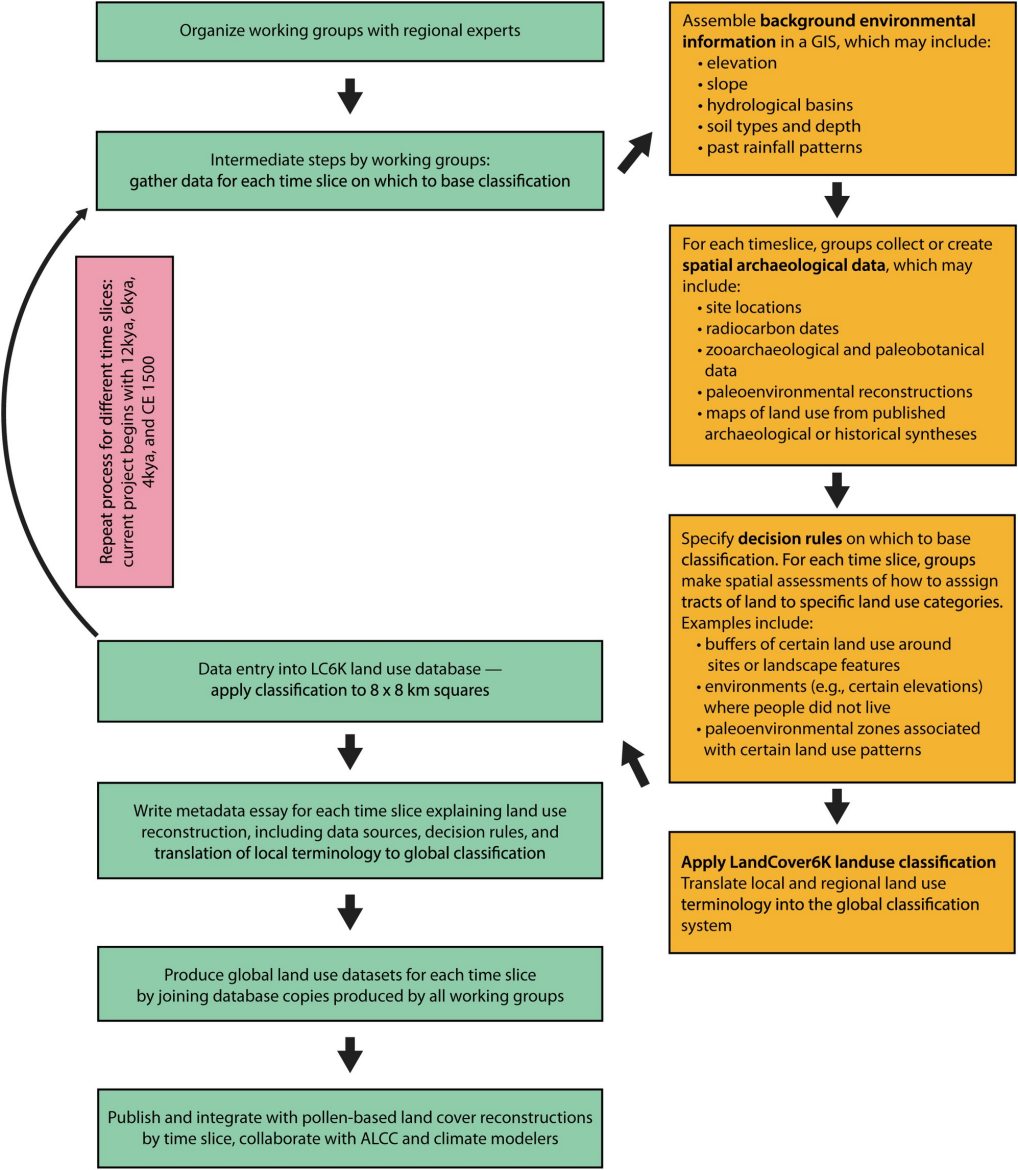
Land Use classification flowchart showing the generalized processes LandCover6k is using to translate regional archaeological data into a spatial format using the classification scheme and the accompanying geospatial database described in the paper.

Each regional group selects, assembles, and uses its own body of 1) background environmental information, 2) spatial archaeological data, and 3) decision rules on which to base their classification. Background environmental information commonly includes raster and vector datasets representing elevation, slope, hydrological basins, soil types, soil depth, past rainfall patterns, and past coastlines for the time slice in question. Regional-group-provided base maps are particularly important for dealing with the important issue of sea level change, which affects which sets of squares will require land-use assessments at each time period. Spatial archaeological data commonly include site locations with information on paleobotanical and zooarchaeological identifications (if available), radiocarbon dates, paleoenvironmental reconstructions, and maps of land use from published regional archaeological syntheses. Decision rules on which to apply classifications include, but are not limited to, spatial assessments such as buffers representing hinterlands around known sites, elevations above which people did not live or practice a certain type of land use, and paleoenvironmental zones associated with a particular type of land use. The generalized processes of translating regional archaeological data into a spatial format using the classification scheme and the accompanying geospatial database are set out in the flowchart [Fig 9].

It is unfeasible to record metadata and references within the spatial database because the database is structured around individual 8 x 8 km squares and most references and data decisions apply to broader areas. Additionally, the recording of references in the spatial database would provide limited space for reviewing arguments in existing literature and does not directly facilitate publication of the resulting maps. Instead, regional groups record references and data decisions in an essay format. Regional groups prepare one metadata essay for their region for each time slice. The essays identify published sources of maps and information used in preparing the LandCover6k digital land-use map and specify spatial “rules” or other logic that were used in assigning grid squares and groups of grid squares to particular land-use categories and land-use variables. Below, we provide an example of this approach for the Middle East at 6 kya.

### Archaeological land use mapping: Mesopotamia and Arabia at 6 kya

**Fig 10:**
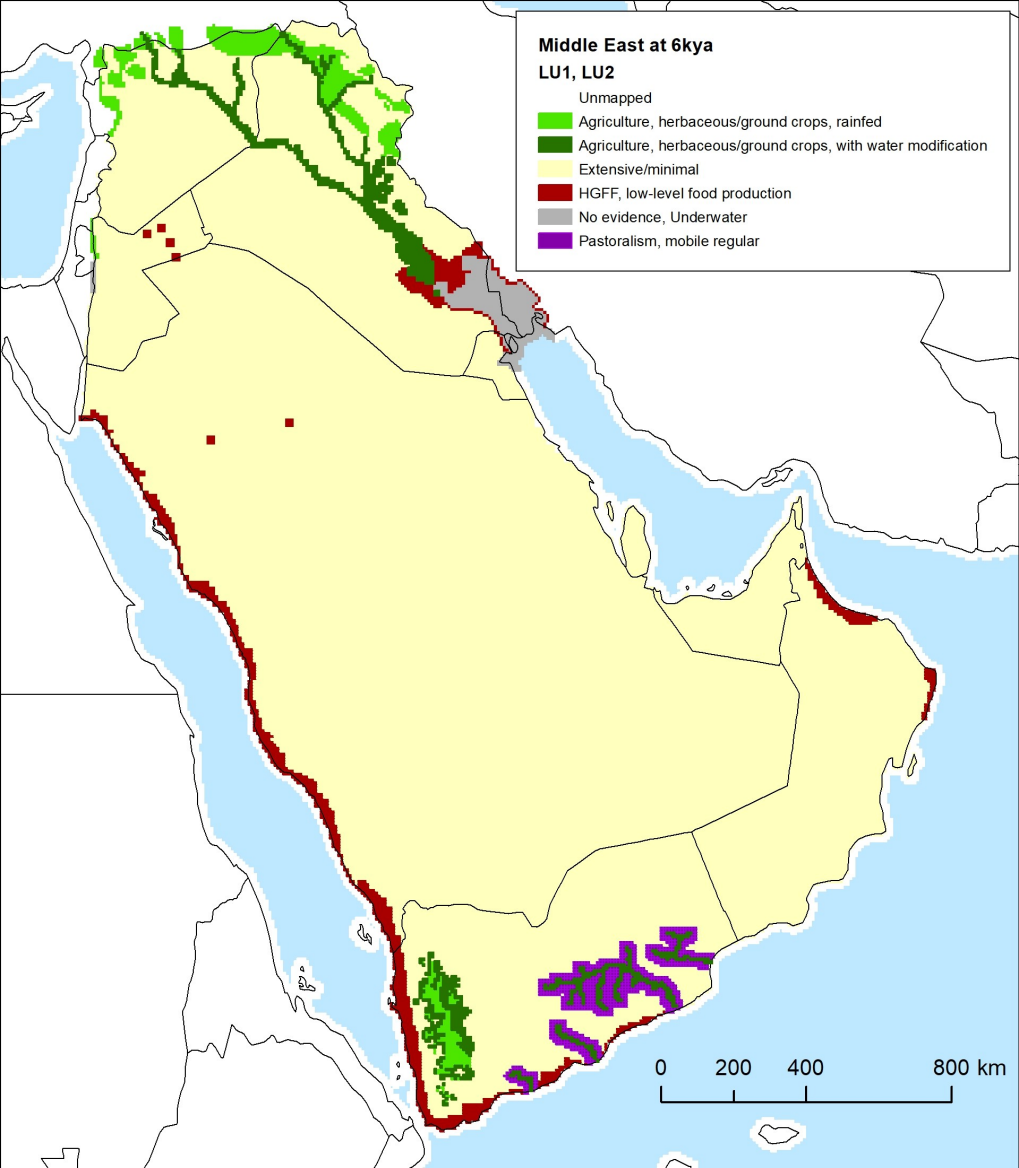
Mesopotamia and Arabia land use at 6 kya, an example using the classification scheme, geodatabase, and classification processes outlined in the paper. Explanations for how the classification has been applied and citations for archaeological data used in this example are discussed throughout the text.

Here we provide a brief preliminary example of the application of the classification system for Mesopotamia and Arabia at 6 kya (Fig 10). Chronological precision of archaeological data vary significantly, and for this time -slice, addition of a temporal buffer means that evidence from 4250–3750 BCE is included within the 6 kya slice. In terms of modern geopolitical boundaries, the example covers the countries of Iraq, Syria, Jordan, Kuwait, Saudi Arabia, Qatar, Bahrain, the United Arab Emirates, Oman, and Yemen. For the purposes of keeping the example brief, we explicitly exclude Iran, Turkey, Lebanon, and Israel, as the coastal Levant and the Taurus and Zagros Mountain zones to the west, north, and east are complex zones that will be addressed in a later paper. The Mesopotamian portion of this exercise shows an example of land-use classification in a zone that is relatively well-studied archaeologically, while the Arabia portion shows an example of land-use classification in a less well-known zone with patchy data coverage and areas that are relatively archaeologically unknown at this time period.

#### Mesopotamia land use data

Data sources for southern Mesopotamia are based on the regional-scale archaeological surveys by Adams [109–111] and Wright [112], as well as paleoenvironmental data. Data sources for northern Mesopotamia are based on regional-scale surveys by several teams working in northern Syria, and more recently, northern Iraq. Syrian data are frequently systematic and well-synthesized in review publications [13, 111, 113], but more recent data from Iraqi Kurdistan have been collected from preliminary reports [114–120]. For southern surveys, the 6 kya time slice corresponds to the Early Uruk period (c. 4000–3500 BCE), when irrigation agriculture and animal husbandry had been well established in the area for more than two millennia. For northern surveys, this time slice corresponds to the Late Chalcolithic 2 and 3 period (c. 4200–3900, 3900–3600 BCE). Many regions of the north were among the early centers of plant and animal domestication in the Neolithic and therefore had a 3000-4000-year history of food production by this time.

The landscape of southern Mesopotamia during this period differed considerably from the present. The Persian Gulf and its associated marshes extended further to the north at this time, reaching their greatest extent around 4550 BCE [121–125]. The land use patterns likely associated with coastline and marsh areas have been mapped according to reconstructions of the spatial extent of these environments in Algaze [126 Fig 1, pp. 202,based on 127]. The Tigris and Euphrates rivers have continuously modified their courses through time via avulsion. Since the advent of irrigation agriculture, human communities have both slowed and accelerated this water course change in different areas [128]. In the third millennium BCE and later, a denser recoverable settlement pattern and surface topography makes it easier to reconstruct the location of river channels and canals [111, 129]. For earlier periods, the reconstruction of watercourses is more hypothetical. We have used hypothesized major watercourses of the late fifth and early fourth millennium BCE as reconstructed in [126, Fig 1, pp. 202; based on 121], the location of known Early Uruk sites from major survey areas, and models of early irrigation agriculture as a basis for assigning 6 kya irrigated agricultural land. Wilkinson et al. [130] argue that early irrigated agriculture in southern Mesopotamia was not widespread, but instead only took place on river levees, which are on average 5 to 6 km wide. It is widely recognized that the major southern Mesopotamian surveys mainly focused on areas in the center of the Tigris-Euphrates alluvium that were largely outside the boundaries of twentieth century agriculture, as ancient sites were more visible in these areas. Ancient settlement patterns nearer the modern courses of the rivers, especially the Tigris, remain poorly documented in grey literature and in local languages, and are, for the purposes of this example, not included here [131, 132]. Additionally, remains of early periods like the Uruk period tend to be deeply buried in some parts of the plain, either as a result of later occupation or alluviation. Thus, our use of known Early Uruk sites in the land use classification surely underestimates the extent of irrigated agricultural land at 6 kya.

In northern Mesopotamia, the river courses have been more stable through time and present-day courses are close to those of several thousand years ago. Rainfed agriculture has been practiced across non-riverine steppe areas receiving more than 200–300 mm of rain per year; irrigation is much rarer than in the south and was almost certainly absent in the 6 kya timeframe [128]. Changes in rainfall, and therefore changes in the spatial limits of where rainfed agriculture is possible, are the main environmental differences for consideration in mapping past land use. We have used the location of Late Chalcolithic sites, and especially the mapping of Late Chalcolithic ‘core’ agricultural areas by Wilkinson et al. [113 Fig 17, pp. 77], as the basis for assigning 6 kya rainfed agricultural land. Such ‘core’ areas have been defined in opposition to a ‘zone of uncertainty’ located to the south and east with lower rainfall. While this zone of uncertainty became an area of settlement and pastoral activity in the Late Chalcolithic 4–5 and especially the third millennium BCE Early Bronze Age, Late Chalcolithic 1–3 settlement was sparse or absent. The rainfed parts of Syria are among the best-surveyed zones of the Middle East, and some additional agricultural zones beyond those identified by Wilkinson et al. exist, for example around the western Syrian site of Ebla [133]. Additionally, recent survey in Iraqi Kurdistan has documented dense Late Chalcolithic settlement in a number of areas that were also ‘core’ agricultural zones at this time, including the Eastern Upper Tigris region (Erbil, greater Zab, Mosul, and Dohuk plains) and intermontane valleys of the Zagros (Shahrizor and Rania Plains) [114–116, 118–120, 134–136]. It seems likely that future research will demonstrate that significant Late Chalcolithic rainfed agricultural settlement extended over most of the major plains in Iraqi Kurdistan, and thus our classification includes them, even though not all have yet been investigated.

Zooarchaeological and paleobotanical data is virtually non-existent for the south for this period because almost no Early Uruk sites have been excavated and political conflict in the region over the last several decades has hindered the application of scientific approaches to sites of all periods. Zooarchaeological and paleobotanical studies in northern Mesopotamia typically focus on the later phases of the Late Chalcolithic period (beginning c. 3700 BCE), when Uruk populations and material culture spread into the north from the south. Based on these studies it seems that some indigenous Late Chalcolithic communities in the upper Euphrates and Tigris basins may have focused on cattle and pig pastoralism, and that intensive sheep/goat pastoralism was introduced later with the Uruk Expansion [137]. However, a recent meta-analysis of zooarchaeological data in the region concludes that sheep and goat were the dominant taxa during both the fifth and fourth millennia, with herd composition tied to precipitation patterns [138]. On the basis of studies in northern Mesopotamia and analogy to later periods in the south, we assume that irrigated cereal, legume, and date-palm agriculture as well as sheep/goat, pig, and cattle pastoralism were practiced in various environmental zones in the south and that rainfed cereal and legume agriculture as well as sheep/goat, pig, and cattle pastoralism were practiced in the north. In the absence of empirical data for southern marsh lifeways at the time, we rely on ethnographic analogy to suggest that communities living within marsh environments practiced some pastoralism and perhaps flood-recession agriculture, but primarily relied on fishing, birding, hunting, and gathering for subsistence [139].

#### Mesopotamia classification

Using the LU classification outlined here, in the area of Southern Mesopotamia (south of Baghdad), the land use for the 8 km pixels surrounding Early Uruk sites and hypothesized late fifth-early fourth millennium BCE river courses have been assigned as LU1 agriculture with LU2 herbaceous/ground crops and canals/channels for water modification. Residents of this region kept domestic sheep/goat, cattle, and pig and grew wheat, barley, pulses, and date palms; these are recorded in the crop and animal variables. In the reconstructed marsh areas, LU1 hunting-gathering-fishing-foraging with LU2 low-level food production has been assigned. People living here may have kept cattle as their main animal domesticate, since pigs and sheep/goat do not thrive in wetland environments. As the extended Gulf area was at this time underwater, it was coded LU1 as no human land use. In the areas outside the marshes and riverine areas, LU1 Extensive/Minimal was assigned; although there is little archaeological evidence for occupation, we presume that people did move across this region.

In northern Mesopotamia (north of Baghdad) we have assigned LU1 agriculture with LU2 herbaceous/ground crops, flood for water modification, and sheep/goat, cattle, pig, wheat, barley, and pulses to squares in the modern floodplains of the Tigris and Euphrates rivers and major tributaries that flow year-round. An assignment of LU1 agriculture with LU2 herbaceous/ground crops, focusing on sheep/goat, cattle, pig, and wheat, barley, and pulses was given to the steppes outside the modern floodplains of the Tigris and Euphrates and in areas interpreted to be Late Chalcolithic agricultural cores on the basis of settlement patterns and excavations (these areas would have received at least 200-300mm of rain per year, and were coded “rainfed” for water modification). This form of agriculture does not necessarily extend into the mountain foothills at the edge of Mesopotamia in Turkey, Northern Iraq, and Iran, though as noted, some Zagros valleys in Iraqi Kurdistan do have dense agricultural settlement at this time. The intermontane valleys within Iraq were defined according to topographic slope using the GTOPO30 digital elevation model. For the purposes of this paper, our mapping example ends here.

Ceramic production was well-established in both northern and southern Mesopotamia by this time (having begun during the Late Neolithic or Pottery Neolithic, after c. 6000 BCE in the north and c. 5400 BCE in the south), and was accomplished on a large scale in cities and towns. Accordingly, ceramic production is recorded in the pyrotechnology variables.

#### Arabia land use data

In Arabia, we have relied on data synthesized by Magee [140], McCorriston and Martin (for southwest Arabia) [141], and Petraglia et al. (for northern Arabia) [142]. The available data are much more unevenly distributed than in Mesopotamia, and the land mass is much larger and more variable. The land use mapping therefore often relies on evidence from individual sites and a concise general assessment is more difficult to produce. Arabia and Mesopotamia show several broad differences in terms of subsistence and land-use leading up to 6 kya. First, Arabia was not within the native range of the wild progenitors of major animal and plant domesticates. These domesticates had to be introduced from elsewhere, especially Mesopotamia (via boat trade in the Gulf, which began by the late sixth or fifth millennium BCE), the Levant, and perhaps (in the case of cattle) east Africa. Agriculture and pastoralism therefore developed later in Arabia. Faunal remains and rock art indicate that sheep and goat were present from at least the late seventh millennium and cattle were present from the early sixth millennium BCE [143–145]. Second, unlike the Fertile Crescent, of which Mesopotamia is a part, agriculture followed pastoralism. In highland south Arabia, domesticated plants are not evidenced until a millennium later than domesticated animals. Many Arabian populations adopted herding practices in the absence of agriculture and apparently also in the absence of a tradition of wild plant collection and cultivation. In most places, pastoralism appears as part of a broader subsistence strategy focused on hunting and/or marine resources. Third, the Neolithic in Arabia was aceramic.

Declining climate conditions during the late fifth millennium BCE Arabian Late Neolithic led to a “Dark Millennium” c. 4000–3000 BC, during which there were major shifts in subsistence strategies and occupation patterns throughout Arabia [146]. Although there was a brief period of slightly greater humidity at 6 kya, this was sandwiched between two significantly more arid phases [142]. Environmental proxy data from Al-Qunf and Hoota caves indicate that the Indian Ocean monsoon system migrated south to its current position by c. 4000 BCE, ending the Holocene Moist Phase [147, 148]. As a result, most of Arabia no longer received the summer rainfall it had in previous millennia. The effects of this aridity varied by region.

In southeast Arabia, archaeological site distributions indicate that there was a near absence of occupation in the eastern province of Saudi Arabia and in the south along the shores and interior of the UAE [140]. The exception to this is Akab, located on a lagoon in Umm al Quwain [149]. Unlike earlier periods, which showed material connections with contemporary inland material culture at places like Buhais 18, the Akab artefactual material is very different from the earlier, Neolithic material, suggesting that in the fourth millennium there was a fundamental break in the coastal to inland pastoral exchange system seen in the Neolithic. These major changes and a new focus on coastal resources may be related to the region’s generally sparse groundwater resources [142].

More sites were occupied along the coastline of the Gulf of Oman. For example, the cemetery at Suwayh I has material from the end of the fifth millennium [150] and the region has three different lithic facies dating to fourth millennium [151]. Charpentier [152] also found over 50 sites dating to 3700–3000 in the Ja’alan, most in the coastal zone, accompanied by large shell middens. Uerpmann [146] attributes intensification of settlement on the Omani but not Gulf coast to environmental and geomorphological differences—coastal areas in UAE are rarely fed by waters from the Hajjar mountains, but the Omani coast has many large wadis carrying water; that area also still received some limited, scattered summer rainfall. Zooarchaeological data from sites like Ras al-Hamra 5 indicate that sheep, goat, and cattle were herded, though it is unclear whether the pastoral system was mobile or sedentary [146, 153]. The Gulf of Oman coast appears to have provided a “refuge” where populations could mainly exploit marine resources and continue to practice some herding during the climatic deterioration; inland areas seem to be largely empty of occupation at this time [140].

The interior of southwest Arabia did not suffer as much from the climatic deterioration because it provided a greater diversity of exploitable economic niches that were used more intensively for pastoralism and agriculture [154]. Some indications of irrigated agriculture appear in highland Yemen [155, 156]. Barley, wheat, chickpea, and possibly millet were present in late fourth millennium BCE Hayt al-Saud and Jubabat al Juruf, slightly later than the time slice considered here, in areas where terrace agriculture may date back to the fourth millennium BCE [128, 157]. Traces of runoff irrigation are found in lowland eastern Yemen at Wadi Sana, a tributary of Hadramawt, dating to the mid-fifth through mid-fourth millennium BCE. Irrigation here may have developed in response to decreasing precipitation, but the major crops are not known [158]. Overall, Harrower [158] and McCorriston and Martin [141] characterize the lowland inhabitants of Southwest Arabia at this time as “pastoralist irrigators,” using agriculture and cattle pastoralism with seasonal transhumance. Pastoralism was also important in the highlands, primarily cattle but also sheep/goat herding [156].

More detailed data exist for the lowlands of southwest Arabia at this time. McCorriston and Martin [141] argue that between 7–5.5 kya there is evidence of cattle ownership with accompanying tribal markings, perhaps suggesting grazing rights in particular territories. The site of Khawlan, north Yeman, shows evidence for cattle and caprines and Kheshiya, Wadi Sana, has evidence for specialized cattle pastoralism in the form of cattle skull rings and collective feasting events. In the Wadi Sana, there is evidence of landscape-scale burning events, perhaps linked to more intensive pastoralism, around 5880 BP [159].

The number of known sites in the Tihama (Red Sea Coast) is small; shell midden sites positioned 5–10 kilometers inland at local environmental interfaces radiocarbon date from the late seventh to the late fourth millennium BCE and therefore extend into the 6 kya time slice. One site in the region had faunal remains of cattle and sheep/goat in contexts with a single radiocarbon date (3600–3180 BCE) falling just later than the time range considered here (SRD-1). This is the earliest recovered evidence for herding in the Tihama [156].

There is substantively less data for northern Arabia and the interior of the peninsula, though recent projects are now providing more detailed pictures of northwestern Saudi Arabia and eastern and southern Jordan [142]. The few sites known from the vast stretches of interior Arabia demonstrate a significant decline in human activity around 6 kya, compared to earlier Neolithic pastoral activity, which had often been concentrated around paleolakes and oases. Surveys in the northern region show significant occupation by the Neolithic from the Saudi highlands through eastern Jordan [140, 160–163] including in and around the Nafud desert [164, 165]. However, many surveyed sites are not directly dated, and it is assumed that they correspond to occupations during more climatically optimal periods than 6 kya. Where dates are available, evidence for continued occupation of the northern region by 6 kya is patchy, and likely significantly less intense in comparison to earlier and later periods.

Settlement may have continued during this time period at the important north Arabian basins of Tayma and Jubbah, around the Nafud desert. For example, the continued presence of the pollen of domesticated plants at Tayma from 6.6 kya [166] onward suggests continued human activity, and a carnelian bead manufacturing site at Tayma dates to this period [167]. Sites in the Jubbah basin mostly remain undated. Petraglia et al. [142] argue that the presence of large, shallow aquifers, and the possibility of rainfall from the winter westerly storm systems at northern oases like Tayma and Jubbah, resulted in more continuity through arid periods than is seen in southeastern Arabia.

Further to the north, in the portions of north Arabia that fall within Jordan and Syria, there is abundant survey data for “late prehistoric” periods that suffers from the same problems noted for the Arabian interior and the Nafud. Sites and features of the Neolithic/Chalcolithic/Early Bronze Age are not precisely dated, but likely mostly date to periods when climatic conditions were more optimal, and few or no radiocarbon dates fall in the 6 kya range. However, there is general consensus among scholars that there was still some low level of continued human occupation of these areas [168]. Betts and Martin [169] have suggested that Chalcolithic sites exist in the *harra* (basalt desert) and *hamad* (limestone desert) of Jordan but that these have frequently gone unrecognized in earlier surveys due to a lack of good diagnostic material. Recent work has, however, identified several sites from northern, southeastern, and eastern Jordan that demonstrate continued pastoralist use of the landscape into the Chalcolithic period encompassing the 6 kya time slice [170–173]. This includes sites like Tell al-Hibr [169], with faunal evidence for a potential mixed subsistence strategy of herding and hunting as in earlier periods. Additional sites like Tulul al-Ghusayn, Khirbet Abu al-Husayn, and Khirbet al-Ja’bariya may have been inhabited year-round [174, 175], the latter of which have radiocarbon dates that overlap the 6 kya time slice [176]. Müller-Neuhof [175] has identified, via systematic survey, evidence for continued seasonal pastoral use, along with flint mining, of the *harra* through this period. While there is good evidence for terrace agriculture in eastern Jordan, at Jawa and other sites, as early as the middle to late fourth millennium BCE [174], this was likely not possible earlier because of the increased aridity in the region. Environmental proxy data for this more northern area is inferred from precipitation-induced changes in the water levels in the Dead Sea [177], speleothem data from the Soreq cave near Jerusalem [178], and pollen cores from the Sea of Galilee [179]. North of the *harra*, there are a few agropastoral villages identified as Chalcolithic/Early Bronze, including Qarassa, Sharaya, Tell el-Baharia, and Tell el-Khazzimi [180–185], that may have been occupied at the 6 kya time slice, but in the absence of radiocarbon dates these sites have not been included. The situation is different in the Jordan Valley, with evidence from large sites on both sides of the river, and significant quantities of radiocarbon dates identifying villages inhabited across the 6 kya time slice [186, 187]. In Jordan, this includes sites like Tuleilat Ghassul, Abu Hamid, Tell esh-Shuna, [187, 188]. Sites in the Jordan Valley utilized mixed farming and pastoral subsistence strategies that included sheep/goat, pig, and cattle with einkorn and emmer wheat, six-row and two-row barley, lentils, chickpeas, and olive [188–192].

#### Arabia classification

Building from this patchy information, the 6 kya LU classifications for Arabia are as follows. Bahrain, Qatar, the coast and interior of UAE and the eastern areas of Saudi Arabia were assigned LU1 Extensive/Minimal. These areas had been inhabited in the Neolithic, but evidence for inhabitation is mostly lacking in the fourth millennium. The coastal plains of Oman and Yemen (including the Ja’alan and the Tihama) are assigned LU1 hunting-gathering-fishing-foraging with LU2 low-level food production; residents here had a primary reliance on shellfish, but also kept sheep, goat, and cattle, and these are coded in the animal variables. There is disagreement about the extent to which the coastal communities in Oman like Ras al-Hamra 5 were sedentary or mobile in a coastal-winter inland-summer pattern attested in more climatically optimal periods. However, it should be noted that no inland sites have been found [140], and therefore the interior of Oman has also been assigned to LU1 Extensive/Minimal.

Given the limited research in highland Yemen, potential agricultural areas have been mapped based on the characteristics of the two known (slightly later) sites discussed above, the dating of terrace systems, and general reconstruction of pre-industrial agricultural methods by altitude as presented in [128 pp. 187, Fig 9.2]. Following this reconstruction, which shows the altitudinal limits of different sorts of agricultural systems for the Tihama (Red Sea, west) versus Rub al-Khali (east) side of the Yemeni highland, we have assigned the following classifications: 1) squares with significant areas above 2500m ASL (on the east side) and above 2000 m ASL (on the west side) were assigned LU1 agriculture with LU2 herbaceous/ground crops with rainfed in the water modification variable. Terrace systems are found in these areas, but unlike lower altitudes, irrigation was not required. 2) squares with terrain 1500–2500 m ASL on the west slopes were assigned to LU1 agriculture with LU2 herbaceous/ground crops with terrace water modification. 3) squares below 1500 m ASL between the highlands and the Tihama coastal plain were assigned to LU1 minimal-extensive because 6 kya sites are not known from this zone (though this could just be due to a lack of research). 4) squares 2000–2500 m ASL on the east slopes were assigned to LU1 agriculture with LU2 herbaceous/ground crops with terrace-based water modification. 5) squares below 2000 m ASL on the east slopes were assigned to LU1 minimal-extensive; in later periods this was an agriculturally productive area under episodic flood irrigation, but evidence from oases like Ma’rib seem to indicate such irrigation agriculture began in the third millennium BCE. Elevations were derived from the GTOPO30 digital elevation model. For all of the LU1 agriculture zones of highland Yemen, we coded cattle, sheep/goat, wheat, barley, and chickpea in the animal and crop variables, following very limited botanical data. Lowland and interior parts of southwest Arabia within 1–8 km square from major drainages not in the Ramlat as-Sabatayn/Rub al-Khali sand deserts (primarily the Wadi Hadramawt and its tributaries) have also been assigned to LU1 agriculture with LU2 herbaceous/ground crops with dam water modification (following Harrower’s [158] identification of runoff irrigation in Wadi Sana), with the same animal and plant variables recorded as for highland Yemen (in the absence of crop data). For these wadi areas, the presence of seasonal mobility and landscape-scale burning are noted. Areas within 2–8 km squares from these wadis are assigned to LU1 Pastoralism with cattle and sheep/goat coded into the animal variables.

All of interior Arabia was assigned LU1 Minimal/Extensive. This classification may become more spatially nuanced in the future with more research, but it also may not, as 6 kya was a difficult period to live in this region.

For northern Arabia, areas around most of the few known sites dating to this period have been assigned LU1 hunting-gathering-fishing-foraging with LU2 low-level food production. This includes the Tayma and Jubbah basins as well as 3 x 3 square (24 x 24 km) neighborhoods around known sites east of Jawa and other sites in Jordan noted above. Communities in these areas may have practiced some limited cultivation of crops like the six-row hulled barley, einkorn, bread wheat, and emmer found at slightly later sites [174, 193]. They commonly herded sheep/goat and possibly cattle [174, 194]. However, they also relied significantly upon highly mobile hunting and gathering, and in places like the Nafud they did not leave architectural remains. Given the research difficulties discussed above, this mapping may under-represent the extent of human activity in the area at 6 kya. Ongoing and future work in the region may clarify this. Areas outside of those surrounding these known sites have been classified as LU1 Minimal/Extensive. For the Jordan Valley, squares immediately adjacent to the Jordan River between Teleilat Ghassul (just north of the Dead Sea) and Tell esh-Shunah (at the confluence of the Yarmuk and Jordan rivers) have been assigned LU1 agriculture with LU2 herbaceous/ground crops with rainfed in the water modification and wheat, barley, pulses, olive, sheep/goat, pig, and cattle coded in the plant and animal variables.

## Conclusion

There is a critical need for data-based global assessments of past human land use. Past land use practices transformed land cover in complex and variable ways and with differing degrees of intensity. Although we know that regional-scale transformations in vegetation and even landforms were sometimes very dramatic, it is not yet clear how significant the aggregate of the many local records of landscape transformation documented by archaeologists, paleoecologists, and historians might be on a global scale. Archaeology and other historical disciplines have generated vast quantities of information over the last century or more, but until now these data have not been made commensurate, nor have they been aggregated at a global scale. Data harmonization of this sort requires a common analytical language, shared categories, and shared data formats, requirements addressed by the development of this classification and the accompanying database. As discussed above, the classification, land use variables, and database reported here are the outcome of an extensive process of consultation and co-design [8] between climate modelers, paleoecologists, and archaeologists. Although purpose-built for the objective of improving climate models [37], this database also has significant potential to inform historical research. Indeed, in some world regions, the syntheses we are building are the first and often the most systematic effort to integrate existing large but scattered and inconsistent archaeological data sets.

Classifying and documenting past land use practices is only one step in understanding the impact of our species on the earth system. While data on land use practices such as plowing, large-scale burning, or flooded-field farming that directly affect chemical cycles, such as carbon and methane, may be immediately relevant to climate models, the impacts of land use histories on anthropogenic land cover change (ALCC) are importantly mediated by climate, prior conditions, and other factors. We therefore adopt multiple approaches to improving ALCC estimates for the past. First, we aim to link pollen-based vegetation reconstructions with archaeologically-based land use data [37]. Pollen based reconstructions, though produced at lower resolution, provide an important test of the land use database [8]. Second, we aim to improve existing ALCC models, which differ significantly from one another [15].

This work is already underway. Harrison et al. [8] outlines a protocol for evaluating the land use and land cover models created from the various archaeological data used in ALCC modeling. It suggests ways that the reconstruction created from archaeological data can be implemented into global land use and land cover scenarios, and how these should be evaluated using independent pollen-based reconstructions of land cover and climate. As part of this, the improved models can then be used in paleoclimate simulations. Within the PMIP (Palaeoclimate Modelling Intercomparison Project) the improved models are being utilized to quantify the magnitude of anthropogenic impacts on climate through time, and in doing so the LandCover6k project is playing a vital role in improving the realism of Holocene climate simulations [8].

While there are multiple approaches ongoing to improve ALCC models [29], LandCover6k is novel in important ways. The structure of our database ensures that data is recorded in relatively fine-scale, comparable spatial units. The classification scheme resulted from an extended workshop process in which archaeologists came together to develop a shared understanding that is built into the detailed descriptions and definitions we have provided. This facilitated the consistent use of classification terms and the assessment of variables in comparable ways. Finally, the classification scheme, database, and resulting products are explicitly designed to both facilitate the work of modelers and to serve as a resource for archaeologists.

Existing efforts to quantify ALCC rely on estimates of past population to inform projections of potential land cover impact [9, 11], and a recent contribution compiled published regional estimates of per capita land use for cropland and pasture as an additional parameter for population-based models [10]. While population is clearly an important factor in the area of land needed to meet resource requirements, there are multiple strategies for obtaining specific resources and thus many different vegetation-change outcomes that may result. Further, population effects are mediated by varied forms and levels of consumption [195]. Demography with an assumption of constant per capita cropland use ignores factors such as surplus production, waste, and agricultural intensification [2]. Existing ALCC models rely on algorithms to, for example, distribute population evenly across the landscape or to distribute past population through linear extrapolation from modern demographic patterns; both of these approaches miss well-known locational preferences of groups practicing different forms of land use as well as ways these locational preferences have changed through time with technological, environmental, and cultural change. Our datasets can thus provide empirically-based corrections and constraints on ALCC models, enhancing their value to earth system modelers.

The classification scheme and accompanying database outlined here is a critical part of this. The classification scheme is a simplification of what were complex systems of land use, but such simplification is necessarily for global-scale aggregation. By coding some variables (domesticates, burning, tillage, etc.) apart from the land use categories, we attempt to both capture commonly-recorded archaeological variables significant to environmental change and also to keep open the possibility of analyzing associations among classes and variables. For example, raised fields are often found in areas under maize cultivation, but our data structure allows this association to be tested and for new combinations to be identified if they exist. We thus highlight the value of this exercise to archaeology and history as well as to earth system science. Archaeological analyses of past land use rely on a very large range of indicators, and data coverage and quality vary significantly across space and time. Our database is designed to be public, iterative, and correctable, able to integrate new data and understandings. As a first step in the harmonization of archaeological land use data, it is necessarily preliminary. Although we begin with a limited number of time slices, there is the potential for more powerful transient time series analyses in the future. The LandCover6k research will be able to identify areas or times of rapid land use change, areas of contention that require further work, and areas that are lacking data but clearly are importantly for land use and land cover research. Applied research and modeling groups associated with ecosystems services such as ARIES (Artificial Intelligence for Ecosystems Services) will also benefit from the LandCover6k work, and from the consistent language used in this classification scheme.

Human impact on the earth system has a long history, but we cannot accurately assess its significance without global-scale synthesis. Past human populations levels, while important, do not directly index past human impact; just as in the present, some groups of people consumed more and/or different resources than others, complicating demographic effects. Historical land cover ‘footprints’ were mediated in part by forms of land use, from gathering and hunting to agriculture and industry. Although the significance of anthropogenic land cover change is widely recognized, existing efforts to model these changes on a global scale are problematic, with competing models varying significantly. Despite the existence of significant archives of archaeological and historical data, these data have not, to date, been systematically used to correct or constrain ALCC models. We have developed a common language for land use classification, a database for recording land use assessments, and strategies for data management and coordination as a first step toward using these important but scattered, uneven, and regionally-focused historical archives to contribute to a better understanding of the earth system.

Figs 2–7 were created using Biorender.com. The authors are grateful to Ka Ki Jacqueline “Jacky” Chan for her help in creating Fig 9.

## Supporting information

S1 File (ZIP)

S2 File (DOCX)
