# Supplementary material for: Mapping past human land use using archaeological data: A new classification for global land use synthesis and data harmonization
Source: PLoS One. 2021 Apr 14;16(4):e0246662. doi: 10.1371/journal.pone.0246662 (PMC8046197; doi:10.1371/journal.pone.0246662)
Supplement: S2 File — (DOCX) [file pone.0246662.s002.docx]

**S2 File.**

Land Use Classifications

LU1: No Human Land Use

This category is narrowly defined as locations for which there is a demonstrable lack of human inhabitation. It is reserved for areas where the preponderance of evidence points to a complete absence of humans. No human land use should also be used for land that was underwater during the time period in question due to sea level change or other hydrological changes. This category should not be used if people are in the general area. If people are in the general area, then the extensive or minimal land use category should instead be used.

Examples: Uncolonized island systems, high elevation areas, or extreme environments such as Antarctica; zones that were under water due to sea level or other hydrological changes (e.g., southern Iraq under the Persian Gulf at ca. 6 kya, see main text).

There are no LU2 classifications for this category.

LU1: Extensive/Minimal Land Use

This category is used for land in which humans are assumed to be present but in a scattered or transitory way that is typically not part of their major subsistence system. The category is best defined through the series of examples listed below; most of these are areas that had roads or routes through which people traveled between other more intensively used locales.

*Examples:* Mountain ranges with access routes or evidence of exploration but not inhabitation (e.g., the Ice Man in the Alps); deserts surrounding oases; wetlands without evidence for habitation; sacred natural areas; burial grounds; conservation areas.

There are no LU2 classifications for this category.

LU1: Agriculture

This category describes the cultivation of domesticated crops exclusive or inclusive of the rearing of livestock and use of wild resources [1]. The category incorporates all forms of plant cultivation involving active human participation, including planting, breeding, crop protection, and harvesting. It includes ‘mixed farming’ and ‘agropastoral’ systems in which domesticated animals are maintained alongside plant crops. We also include livestock-based systems (e.g., dairy or meat farming) where herbaceous plants are cultivated and/or managed for grazing within restricted pastures. However, specialized pastoralism is afforded its own LU1 category (see below). Agriculturalists often also engage in some hunting and collection of wild resources; these activities are implicit within the category. Specific plant and animal domesticates are coded separately from this category. Modes of water provision and landscape modification affecting productivity (e.g., whether cultivation is rainfed or irrigated; tillage; manuring) should be specified via the additional variables (see section on land use variables below).

LU2: Herbaceous/ground crops

Herbaceous (non-woody) ground crops are generally managed according to regular cycles, which may be seasonal or multi-annual. This includes grass pastures maintained for livestock farming. Field or garden systems are permanent; though fallow periods are included, this is distinct from shifting systems that employ longer cycles of exploitation/recovery (e.g., swidden/shifting systems, see below). Water needs are met through rainfall or irrigation (defined as a separate variable); a distinction is maintained for systems exploiting flooding or waterlogging. This category is the closest to what models such as HYDE 3.2 [2] designate as ‘cropland.’

*Examples*: Agrarian systems in medieval Sweden [both fields and pastures, 3]; West African sorghum/cowpea intercropped fields [4]; Central European LBK Neolithic small garden plot agriculture [5]; Nile farming complexes [6]; Cereal and legume cropping alongside pastoralism in the Fertile Crescent of the Middle East [7]; Quinoa agriculture in the Southern Andean highlands [8].

LU2: Swidden/shifting

Cultivation occurs in a cycle in which a land unit is cleared of existing vegetation – the latter often burned in swidden systems – before being subject to one or several seasons of crop growth followed by multiple seasons of fallow to restore fertility while cultivation ‘shifts’ to a different location [9–11]. The extensive literature on swidden/shifting cultivation has highlighted the variability of such systems. As Mertz, et al. [12] explain, “…the diversity of land and resource management denominated by the term swidden makes it difficult to provide a widely applicable definition. There is little agreement on the term, although swidden cultivation, shifting cultivation, and slash-and-burn agriculture are often used synonymously, despite the meanings not being identical. Swidden is a word of Scandinavian origin meaning "land cleared by burning", but fire-free, mulch-based systems exist on Pacific Islands and elsewhere and are more appropriately termed shifting cultivation. Slash-and-burn is often used for a wide range of land use practices where no shifting of fields takes place.” Our classification groups these practices together. For Southeast Asia, Mertz et al. [12] define swidden “...as a land use system that employs a natural or improved fallow phase, which is longer than the cultivation phase of annual crops, sufficiently long to be dominated by woody vegetation, and cleared by means of fire.”

Fire use is coded separately in our analysis and need not be present to use this land use category. Key distinctions between swidden/shifting cultivation and herbaceous ground crops relate to cropping cycles and to the mixture of herbaceous and woody plants typical of most swidden systems.

*Examples*: Mexican and Central American *milpa* cultivation [13]; *Citemene* in Zambia [14,15]; *Ladang* in Indonesia [16]; *Jhum* in northeastern India and Bangladesh [17].

LU2: Wet cultivation

This category comprises temporarily or permanently wet or waterlogged environments exploited for cultivation. While conventional irrigation technologies like canals and dams might be employed, wet cultivation is distinctive in its association with waterlogged conditions. Strategies might be aimed at enhancing the retention or drainage of water, often through constructions like raised fields, pondfields, and paddies. In order to be classified as wet cultivation, fields should be wet for part of the growing season. Flooded fields such as rice paddies are typically drained for a portion of the growing season and may be dry when not in use; farming carried out in seasonally flooded areas may not entail year-round moisture. Still, in these situations the wet or waterlogged state of the ground is a critical factor. The long-term use of wet agricultural practices can sometimes lead to distinctive landscape modifications such as the formation of paddy soils [18] or regionally altered landforms [19]. Because wetlands (both natural and constructed) are the single largest source of global atmospheric methane emissions [20], mapping wet cultivation has special importance. The mere presence of irrigation, which can be coded as a separate land use variable, does not require use of this category – for example many Medieval and Early Modern millet and cotton fields in southern India were irrigated by runoff-fed reservoirs [21,22] and would not have had standing water or saturated soil for an appreciable period of time; they therefore would be classified as herbaceous ground crops.

LU3: Rice paddy / taro pondfields

Channels feed water-filled basins enclosed by earth or stone bunds creating aquatic environments for the cultivation of hydrophytic crops such as taro or rice. Water in rice paddies is typically flowing rather than stagnant and these locations may contain standing water for anywhere between a few months to year-round.

*Examples*: Asian rice paddies [21, 23]; Taro pondfields in Polynesia and Melanesia [24,25]; West African rice paddies [26–28]; *Masakwa* in the Lake Chad Basin [26,29,30].

LU3: Raised Fields / Chinampas

Soil is excavated and piled within designated areas so that cultivation surfaces are elevated above the level of water bodies, permanently or temporarily/seasonally, creating networks of cultivation surfaces [31]. The practice is used to regulate or enhance drainage and soil nutrient levels. There is a wide range of morphological variation within this category, but in general, raised fields are presumed to be permanent or semi-permanent and to have been under cultivation at the time period under consideration.

*Examples:* Mexican *chinampas* [32]; Amazonian raised fields [19]; Central African raised fields [26].

LU3: Wetland cultivation

Cultivation of naturally waterlogged environments, such as wetlands, marshes, and swamps. This can include the exploitation of seasonally flooded areas if cultivation is explicitly related to waterlogged conditions. This category does not typically include natural basin agriculture in floodplains where cultivation takes place after recession, unless water is deliberately/mechanically retained. However, the category does include recession agriculture in wetland environments like river deltas.

*Examples:* Iraq marshes [33]; Inland Niger delta (*crue-décrue*) [4,34].

LU2: Agroforestry/Arboriculture

Agroforestry and arboriculture are land use practices oriented around the use of woody perennials intentionally introduced or maintained within an agricultural land management unit [35,36]. Because past land use often involved the use, manipulation, and even creation of areas dense with trees and other woody plants, it is not always possible to distinguish between agroforestry and arboriculture. These two systems are distinguished by the relatively greater importance of non-woody crops in agroforestry systems and the more focused use of tree crops in arboricultural systems. Terrell, et al. [37] focus on this dimension of diversity in their definition: “The term “agroforestry” refers to predominantly arboreal-based economies in which manipulating and maintaining forest ecosystems and forest resources—including birds, reptiles, amphibians, insects, mammals, roots, grasses, leaves, and a wide range of medicinal plants—are central.”

The productivity and sustainability of agroforestry systems are reliant on dynamic ecological interactions between trees and crops and/or livestock [10,37]. The function of tree retention can include soil conservation and enrichment or the provision of shade and windbreaks, as well as secondary products such as timber and foods (e.g., nuts and seeds). Livestock can suppress weed growth and enrich soils via dung. The range of species present in an agroforestry unit can approximate that of natural forest, but can also be monocultural, such as in coffee agroforests [11]. Arboricultural systems include the cultivation and cropping of woody species; this might include orchards, olive groves, wine cultivation, banana plantations. Agroforestry and swidden cultivation may be difficult to distinguish; in many swidden systems trees are planted or retained within cultivated areas, for example. The agroforestry category should be used if it is clear that woody plants are consistently maintained in planted areas and if woody plants dominate cropped areas.

LU3: Arboriculture

Cultivation and cropping of trees or woody plants either in single or multi-taxa associations and tree crops that may be either wild or domesticated species. Arboriculture is distinct from the harvesting of wild fruit and other tree resources, including wood. Orchards and tree plantations are common forms of arboriculture; these may include non-woody components but should be primarily composed of tree crops. A few non-woody crops such as bananas or other palms may be grown as orchards, and thus would be included here. The cultigen codes discussed below would prevent these areas from being analyzed as woody plants.

*Examples*: olive groves [38]; date palm gardens [39]; apple orchards, (wild) oil palm arboriculture as in the Late Stone Age Kintapo Tradition, Ghana [40]; early Holocene fruit groves and arboriculture in island Southeast Asia [41].

LU3: Agroforestry with soil enrichment

Soils associated with an agroforestry unit are a product of indigenous soil management, as detailed by Gourou [42]. This includes practices designed with the aim of soil improvement as well as those where enrichment is an unintentional by-product of habitation or other use [43,44]. However, enriched areas are vital to productivity and the anthropogenic nature of enriched soils such as the Amazonian Dark Earths (ADE) is not in question [e.g., 45]. Typically, ‘dark earths’ are rich in soil organic matter (SOM), nutrients, and biochar (pyrogenic carbon) [46]; they often contain artifacts such as ceramics and animal bones and, as the name suggests, are typically dark in color.

*Examples*: Amazonian Dark Earths (*terra preta/mulata*) [47–49], African Dark Earths [43,44]; Kilimanjaro forest gardens [50].

LU3: Agroforestry without soil enrichment

Soils associated with agroforestry units are not visibly differentiated from those of unexploited areas, although there may be elevated levels of organic matter and/or of elements such as phosphorus and potassium [51].

*Examples*: Regions clearly under agroforestry in the past, but without evidence for dark earths, or dark earths are very poorly developed; Pre-contact Rekohu (Chatham Island), New Zealand [52].

LU1: Hunting-Gathering-Fishing-Foraging

“‘Hunting and gathering’ is a conventional term for subsisting on the ‘natural produce’ of the earth by hunting and trapping wild animals; gathering wild roots, fruits, and berries; fishing; and collecting honey of wild bees, as opposed to producing food by agriculture and herding.” [53–58]. We take “forager” as a synonym for “hunter-gatherer” [59]. This broad category uses an expanded definition which also includes nut, seed, and vegetative plant food collection and small-scale cultivation of wild resources. In some cases, this can include use of small-scale domesticates as, for example, among Amazonian communities who are primarily focused on gathering and fishing but who have limited amounts of maize cultivation. Many historically and ethnographically documented hunting and gathering groups practice some form of management of wild plants and/or animals, altering landscapes and creating what Lee [60] refers to as “engineered niches.” Since the line between exploitation and production of a resource can be ambiguous, the existence of some level of resource management should not disqualify an area from being coded as HGFF. Similarly, the use of wild plants or animals in association with agriculture is very common [e.g., 61–64] and should not disqualify an area from being coded as agricultural. Many groups forage and also fish. Various forms of aquatic resource exploitation are included in this category and are specifiable at LU2.

LU2: Hunting-gathering-foraging only

Land use is dedicated to the procurement of wild resources with no significant protective management or cultivation (i.e., no low-level food production). Hunting includes fishing, though extensive use of aquatic resources should be classified ‘LU2 Broad-based and aquatic resources’ or ‘specialized fish production’.

*Examples*: Hazda, East Africa [65]; Pre-1863 Great Basin Shoshoni groups; North America [except the Owens Valley Paiute, with low-level food production 66,67]; Many terrestrial economies pre-dating domestication.

LU2: Broad-based and/or marine/aquatic resources

Exploitation of a wide range of terrestrial wild and marine/aquatic resources. This category is distinguished from the above by larger groups and therefore the potential for more concentrated impacts on land cover. While communities may or may not be sedentary, this land use type denotes generally low mobility among inhabitants. As such, local-scale impact on vegetation might be expected to be significant, despite the fact that agriculture is not present. Archaeological indicators of this category might include shell middens and/or evidence for large-scale processing of wild plants such as acorns, large grained grasses and wild barley, or einkorn and emmer, in conjunction with evidence for large sedentary or semi-sedentary settlements. Many of the land use practices described by Price and Brown [68] in the context of their category of ‘complex hunter-gatherers’ would fit here.

*Examples*: Coastal Pacific Northwest of North America [e.g., 69]; Early Chulmun period, Korea [60]; Natufian groups of the Euphrates Valley, Syria [70], Ertebølle culture Mesolithic, Denmark [71]; Saharan aquatic groups [72,73]; Kansyore communities around Lake Victoria, East Africa [74–76].

***LU2: Low-level food production***

Land use is dominated by the procurement of wild resources but supplemented by activities such as the protective management of desirable wild species, for example wild cereals or selected wild animals [66], or by limited production of domesticated crops and/or possession of domestic animals. This category lies within what Smith [66] calls the “middle ground” between HGFF and agriculture. This distinction is not necessary in our classification as plant and animal domesticates, if present, can be independently coded. This category may include the use of fire to increase the production or yield of wild food resources, such as wild cereals, nuts and berries, and to attract wild animals, such as deer, for hunting [77]. As landscape level burning is also a land use strategy used by farmers to manage land for agriculture (see LU2 Agriculture—Swidden/shifting above), where known to be present, the use of fire to manage land is additionally coded as one of the variables.

*Examples*: Hopewell, Eastern North America [78]; Jomon period, Japan [79]; Pre-domestication cultivation; Swifterbant and Hazendonk Middle Neolithic, Netherlands [80–83].

LU2: Specialized fish production

This category captures the ‘‘production’ or management of fish, especially in constructed fish ponds or fluvial, tidal fish traps. This is a specialized form of production that, in some places, can cover large areas and has been documented from as early as 3500 BCE in China [84]. If both fields and fish ponds are used by the same people, these would be coded differently, because the coding refers to the actual land area and not to the agents of land use (i.e., inter-tidal zones would be coded differently from coastal hinterlands).

*Examples*: Pre-Columbian Bolivian Amazon [85]; Pre-contact Kaua’i, Hawai’i [86].

LU1: Pastoralism

This category covers activities specifically related to the grazing of livestock, involving minimal or no cultivation of plants. This includes land inhabited solely by specialized pastoralists as well as areas in which grazing was undertaken separately from cultivation, such as the hinterland around an agricultural settlement or seasonal mountain pastures. For this category, it is important to keep in mind that the unit of analysis is land, and not the identity of the people using the land. The classification implies a degree of mobility, but this can range from daily schedules of grazing/corralling to full itinerance. The majority of so-called ‘pastoralist’ communities actually practice some cultivation and therefore, within our framework, the areas they inhabit should be classified under the ‘agriculture’ LU1 category, with variations specifiable at the LU2 level under ‘animals’. For example, dairy farming, where some crops are grown to provide animal fodder, would likely be classified as ‘herbaceous/ground crops’ with livestock (e.g., cattle) attributed using the ‘animals’ variable.

LU2: Anchored

This category is based on intensive livestock-based production by a settled human population, such as the grazing of common land by local communities. Since most settled communities would be expected to engage in some form of cultivation, this category is reserved for land where such activities are minimal. Available pasture may not be so extensive as for ranching (see below), and grazing pressures can be intense within a limited distance from settlements. Land use systems combining agriculture and the management of grazing and/or browsing animals are sometimes referred to as ‘mixed farming’ or ‘agropastoralism;’ in our classification these are separated, with areas under agriculture classified as agriculture and areas used exclusively or nearly exclusively (these areas may also be used for gathering or hunting, for example) for livestock as ‘anchored’ pastoralism. As noted, both plant and animal domesticates can be coded independently of land use classification.

*Examples*: Northern European moorlands (e.g., England: Bodmin Moors and Dartmoor; Yorkshire moorlands) [87]; Early Historic to contemporary southern India, village-based agriculturalists with flocks of caprids and bovids [22]; Grazelands surrounding Early Bronze Age agropastoral villages in northern Iraq and northwest Syria, where features called hollow ways show the extent of agricultural fields versus open steppe land [88]; Camelid breeding in the Andean altiplano [89, 90].

LU2: Ranching

Intensive livestock production within large but restricted landholdings. Grazing takes place within a defined area, either enclosed (i.e., fenced) or not. Rangelands [areas under ranching; cf. 19] can be extensive and are often given over exclusively to the purpose of grazing. Areas under ranching typically do not have cultivated pasture grasses or other plants (see herbaceous ground crops, above).

*Examples*: North American West [91]; South American Pampas [92]; Australian Aboriginal land management [93]; Hawaiian ranching [94].

LU2: Mobile – regular

Herding operates according to a schedule of regular intra-annual movement, often dictated by seasonal variability in environmental conditions. Vertical movement in response to changes in pasture availability are one form of mobile-regular pastoralism; these kinds of seasonal rounds characterize forms of transhumant pastoralism practiced in the European, Middle Eastern, and Central Asian mountain ranges like the Alps, Pyrenees, Taurus, Zagros, and Hindu Kush. In other cases, the mobility of pastoralists may be dictated as much by social factors and land access as by seasonality; specialized pastoralists in western India, for example, move large flocks on a regular seasonal round, camping on the fallow fields of agriculturalists [95]. Horizontal movement might also be in response to seasonal rainfall, seasonal flooding of river valleys/deltas, or other temporary resource availability. Where mobile-regular pastoralism overlaps with land under agriculture, the latter should be prioritized, for example in the Inland Niger Delta [96].

*Examples*: Transhumant pastoralism in the Alps, Pyrenees, and Picos de Europa Mountains [97,98], Transhumant caprine and cattle pastoralism in recent centuries across parts of the Middle East, North Africa, and western Central Asia [99], Middle Holocene pastoralism in Eastern Sahara [100,101] and Central Sahara [102,103].

LU2: Mobile – irregular

Herders and/or livestock move in response to environmental or socio-cultural drivers at an intra-annual frequency, with settlements occupied on a temporary or occasional basis, such as among the Peul/Fulani in northern Senegal [104]. These patterns of movement are generally irregular and opportunistic with no repetitive schedule of presence in a particular location. While groups may be ‘tethered’ to a greater or lesser extent to a central settlement or place [*sensu* 105], herders exploit wide and relatively unrestricted rangelands. Where ‘tethering’ applies, outlying camps might facilitate mobility beyond the spatial limits of a daily grazing-corralling regime.

*Examples*: Nomadic groups on the central Asian steppe [106]; African savannah pastoralism, i.e., Fulani, Maasai, Turkana [107–110]; Tuaregs in the central Sahara mountains [e.g., 111]; Dromedary camel pastoralism in the Arabian deserts [112].

LU1: Urban/Extractive industries

In contrast to other LU1 classifications, this category is explicitly linked to land cover as much as land use. It is reserved for areas where human activity has led to land cover being highly modified, essentially free from vegetation. It applies to locations where the majority of the area encompassed can be classified as a built environment. Besides large cities, this classification is extended to include land where vegetation (or potential vegetation) has been removed, such as open-cast mines, quarries, or locations of industrial production, including salt flats used for salt production. Manufacturing industries might also be included here, particularly in the post-Industrial Revolution time slice given the well documented impacts on the biosphere of many such industries [113]. Because the database uses 8 x 8 km grid squares, only very large-scale examples of this land use classification are likely to be registered.

LU2: Dispersed urban/peri-urban

Medium-density population centers with extensive construction, but including space between buildings, such as might be used for food production within the settlement limits.

*Examples*: Mayan cities with evidence for urban farming [114]; Angkor, Cambodia [115]; Medieval Vijayanagara, India [21]; Swahili towns [116]; Andean Late Intermediate ‘pukaras’ [8]; Atlantic Coast kingdoms in West and Central Africa, e.g., Benin City, Ife-Ife, Mbanza-Kongo, Loango, Tio [117].

***LU2: Dense settlement***

Structurally complex, high population-density, and/or extensively paved locations in which the built environment dominates. Cities without extensive areas of vegetation; parks or roadside trees may be present, but not extensive fields or open areas.

*Examples*: Medieval London [118]; Tenochtitlan [119]; imperial Rome [120].

LU2: Mining/Quarrying

Large-scale resource extraction, such as open-cast mining or extensive quarrying, resulting in the removal of existing land cover and pollution of large regions by metallurgic emissions [121,122].

*Examples*: Coal fields of northern Europe/China; Historic tin, copper and gold mining areas in Britain and Ireland; Colonial-era Potosi silver mines of Bolivia [123]; Bronze-Age Kargaly, Russia [124]; Katanga copper mines in the Democratic Republic of Congo [125]; Salt production in Sichuan Basin, China [126].

Land Use Variables

In addition to the hierarchical classification system, the database records information on variables that can be relevant across land use systems. These options provide the opportunity to ascribe greater levels of detail and to add nuance to these simple classifications. For example, a land area classified as LU3 ‘rice paddy/taro pondfields’ could, through the applicatition of variables, code the fact that rice is grown, water buffalo are kept, and land is ploughed, providing a fuller picture of land use without the need for more land use categories. The variables defined in this section are concerned with the specific characteristics of land use within a database grid cell (see main text and SI-2); other variables within the database facilitate evaluation of data quality and data coverage.

Decisions about which variables to include were, like the classifications themselves, based on extensive consultation with a range of scholars and represent a series of compromises between what climate modelers would like to know about the past and the kinds of evidence archaeologists typically collect or are able to collect. For example, although stocking rates of past livestock are of interest because livestock are potentially drivers of global change [127], in situations without historical records, this information is generally not possible to recover. Archaeologists do, however, routinely document the types of domestic animals used and even these data can be useful in the aggregate. Similarly, although past wood harvest rates [e.g., 128] are difficult, if not impossible, to record [but see 129], the well-known association between deforestation and pre-industrial pyrotechnologies, such as iron and steel production, which typically use wood-based charcoal for fuel [130], makes it useful to systematize information on the times and places that the products of these technologies are found. Even in regions with minimal archaeological research, whether or not metal artifacts were made and used is generally known.

Where a variable is inapplicable, unknown, or its relevance undetermined (e.g., whether metal production was practiced or whether metal artifacts were acquired by long-distance trade), the field should be left blank. Multiple (up to 3) options can be selected for each variable except ‘fire,’ where only one option can be selected (“present”, “absent”, or “debated”). Selections should be ordered by prevalence, where possible (e.g., most important first). Land use variables recorded in the LC6k database are not intended to be comprehensive nor to replace existing databases [of archaeological plant and animal remains.](http://www.ademnes.de/)

Cultigens

This category refers to domesticated or near-domesticated plants known to be present in the location and time recorded. While there are many plant domesticates, the database focuses on those plants which may have larger impacts on vegetation cover and land use practices.

Though there may be multiple plant domesticates listed in publications, the database only allows three (ranked) choices from the following list: wheat *(Triticum* sp.*);* barley *(Hordeum* sp.*);* pulses (not including *Phaseolus* sp.)*;* sorghum *(Sorghum* sp.*),* millets (here excluding *Sorghum* sp. and *Eragrostis tef );* maize *(Zea mays);* quinoa *(Chenopodium quinoa);* beans *(Phaseolus* sp.*);* squash *(*Cucurbits*);* olives *(Olea* sp.*);* vines *(Vitis* sp.*);* rice *(Oryza* sp.*);* tubers (a broad category including cassava, potatoes, yams, etc.); teff *(Eragrostis teff);* orchard/tree crops; sugar cane *(Saccharum* sp.*);* oats *(Avena* sp.*);* rye *(Secale* sp.*);* sago *(Metroxylon sagu);* cotton *(Gossypium* sp.*);* other*.*

Animals

This category refers to domesticated or near-domesticated animals known to be present in the location and time recorded. While there are many other animal domesticates, the database focuses on animals commonly kept in larger groups that therefore may have a larger impact on vegetation.

The database allows three (ranked) choices from the following list: goat and/or sheep (caprids, e.g. *Capra* sp.*, Ovis* sp.*);* cattle *(Bos* sp.*);* horse or donkey *(Equus* sp.*);* camel *(Camelus* sp.); water buffalo *(Bubalus* sp.*);* pig *(Sus* sp.); llama or alpaca *(Lama* sp.*, Vicugna* sp.*);* reindeer *(Rangifer* sp.*);* yak (*Bos grunniens*); domesticated fowl such as duck (*Anas platyrhyncho domesticus*), chicken (*Gallus gallus domesticus)*, goose (*Anser anser domesticus*), and turkey (*Meleagris gallopavo*); other*.*

Water and landscape modification

This variable is used to differentiate agricultural systems that rely on ‘natural’ cycles of water or soil nutrient availability (i.e., rainfed or flood-based systems) from those in which interventions are made in order to increase the productivity of a given land unit. Such interventions can be intended to enhance water retention and availability, to enhance drainage in areas where water levels are excessive, or to add nutrients to soil. We focus on manuring, rather than other forms of fertilization, as this is the most archaeologically visible example of such practices. There are many forms of archaeological evidence – from landscape modification to stable isotope analysis of crops – that may allow documentation of this variable.

Variables are: rainfed; flood; canals/channels; terracing; qanats, cistern/reservoir storage; drains/water reduction; manuring; other.

Tillage

Despite the known significance of anthropogenic soil erosion in the global carbon budget [131], there is at present no global-scale synthesis of past human impact on soils except in the recent past (<https://esdac.jrc.ec.europa.eu/resource-type/datasets>), though there have been some attempts to model changes using HYDE [132]. Evidence for soil erosion due to agricultural practices has been estimated in river catchment areas through the analysis of sedimentary deposition rates [133], but this remains limited to particular regions and is not a global methodology or synthesis. One of the ways in which past land use resulted in soil turnover was through tillage. In their research on two agricultural fields in Europe, Van Oost et al. [131] found that tillage-induced erosion and deposition exerted a large influence on soil organic carbon (SOC) redistribution and soil profile change at a time scale of a just few decades, suggesting that longer-term patterns might have an even greater impact. While archaeologists sometimes find direct evidence for tillage such as plow marks [134–136], tillage is more likely to be inferred on the basis of tools such as hoes, plows, and digging sticks [137]; paleobotanical analyses [138–142]; and geoarchaeological evidence [143–148].

Variables are: Digging stick/dibble; plow/ard; tractor; unknown.

Pyrotechnology

This variable is concerned with non-food production processes involving intensive fuel use, whether in the form of wood, charcoal, coal, or dung. As noted, premodern wood harvest rates are of potential importance to earth system processes [149], but these can be difficult to estimate. Locations with large-scale pyrotechnologies may be presumed to use more wood (in the case of wood or charcoal-fired technologies) than those without [150,151]. It is important to note, though, that other uses, such as construction, may have driven large-scale wood acquisition strategies [152]. However, the assumption that all industrial smelting led to deforestation is not warranted, as Eichhorn and Robion-Brunner [153] demonstrate for western Africa in a region with more than 2,000 years of large-scale iron smelting [see also 130,154]. Metal production options refer explicitly to the presence or absence of industrial processes (i.e., smelting) rather than the use of metals. The “large-scale ceramic firing” option is intended for large-scale production; “household-level” ceramic production should not be included.

Variables are: Metal (copper/bronze/tin/silver); Metal (iron/steel); Charcoal; Glass; Faience/large-scale ceramic firing.

Settlement mode

This variable relates to the distribution and character of settlements within a land unit. Although settlement distributions are not necessarily directly linked to the nature or scale of vegetation change in the past, they may enhance understanding of the distribution of human presence. In general, more aggregated settlements might be expected to have a greater impact on regional vegetation than more dispersed settlements, but these associations remain to be investigated. Although climate modelers did not request information on settlement mode, this information is often available archaeologically and may help to contextualize other recorded land use data. While many regions will have more than one mode of settlement present, the most intensive option (variables are ranked from least to most intensive) should be selected.

Variables are:

*Dispersed*: occasional, discrete (non-connected) settlements, either individual dispersed houses or hamlets (small settlements). In an agricultural society with a dispersed settlement pattern, cultivators typically live near their fields, spread out across the region under cultivation.

*Aggregated*: dense settlements. Includes small towns and villages, as well as temporary large agglomerations. In an agricultural society with an aggregated settlement pattern, cultivators typically live in defined villages or towns and move out to field areas to work, in some cases for extended periods on a seasonal basis.

*Non-sedentary*: temporary settlements, such as the camps of mobile pastoralists, HGFF groups, or short-term camps of agriculturalists. Settlements occupied on a sub-annual basis.

*Urban centers present*: Urban centers are present but are of insufficient size to warrant classification of the grid cell as LU1 urban/extractive.

Fire

The use of fire in the landscape has arguably been a major factor in anthropogenic landscape modification [155]. Some HGFF land use practices used landscape-scale burning to clear land of woody vegetation and to encourage new plant growth for wild animals. The vegetation history of both Australia [156] and the Great Plains of North America [157] have been partially structured by anthropogenic burning. A global aggregation of data on fire history is greatly needed, and while the Global Charcoal database [158–161] is under construction, the LC6k database can supplement this with understandings of how burning integrates with other land use variables. Agriculturalists, too, may practice landscape-scale burning (as opposed to the use of fire for cooking, warmth, household-level ceramic production, or other uses we presume were nearly ubiquitous in the past). As noted, fire is a critical element in some swidden/shifting agricultural systems [13]. Historically, fire suppression was a feature of colonial regimes in places such as India [e.g., 162], suggesting that regional-scale burning might have been more widespread in the past than is generally appreciated [see also 163,164]. Seasonality of burning is also an important factor to consider in particular contexts where the data quality is suitable [165–167], as it may provide information on management practices and the impact of these practices on the balance between herbaceous and woody vegetation and on soil carbon sequestration. This will be incorporated once a large-scale mapping effort to assess presence/absence of burning has been achieved.

Variables are:

*Present:* Evidence for deliberate/managed burning of the landscape

*Absent*: Evidence that there was no deliberate/managed burning

*Debated:* No clear evidence for anthropogenic burning, though it might be expected based on ethnographic analogy or other indicators (e.g., increased charcoal in sediment records)

Data Coverage and Quality

There are significant differences in the volume, type, and quality of archaeological data. This unevenness is structured by many factors: preservation, site visibility, scholarly interest, and perhaps most importantly, by time and money invested in research. Countries with well-developed cultural resource management or heritage laws have disproportionally larger datasets and archaeological records that reflect both common research interests as well as the locations of sites threatened by development. The mere presence of large archaeological datasets does not, however, ensure data access. In the United States, for example, each state maintains its own site records and there is no mechanism to ensure that all research, even within the state, will be deposited there. The situation in Europe is similar [168]. Moreover, as noted, past land use is studied via a wide range of sources, including landscape features from terraces to towns, plant and animal remains, tools and technology, and others. The facilities and funding for specialized analysis are not evenly distributed across the globe, so analytical specificity may vary greatly. For example, in a region where charred plant remains are not routinely studied, scholars may know that agriculture was being practiced because they have remains of implements like hoes, but they may not know which crops were used. In another context, scholars may be able to make more precise determinations of seasonality or use isotopic analysis of crop remains to recognize prehistoric manuring practices [e.g., 169]. All these cases relate to past agriculture, but some contexts are more fully studied than others. The classification and database variables were designed to manage this sort of data diversity, allowing very broad categories to be used where more specific information is lacking, but also providing more specific categories and variables where more information is available. To keep track of data diversity, we also include explicit data coverage and quality variables.

In consulting with working groups from around the globe, we discussed data quality at length, developing simple ordinal scales to rank data coverage and quality. One challenge of such a scale is comparability – an ‘understudied’ region in Eastern Europe, for example, may be much better known than a ‘well-studied’ one in another location. The scale distinguishes between ‘coverage’ – the extent to which the land surface has been studied archaeologically – and ‘quality,’ the forms of evidence available. Thus, a region with large-scale surface surveys may have good coverage, but in terms of evidence needed to make land use assessments (well-dated, well-located sites with analyzed plant and animal remains), the quality of the data may be low. In some regions, well-studied locations may be surrounded by little-known areas. Our classification attempts to capture some of this information.

Coverage variables are:

*Coverage high*: many areas have been surveyed and, in general, there is a good sense of where sites of different time periods and types are located. Complete coverage is never possible, but these are areas that can be said to be relatively well-known. Generally, these are regions with cultural heritage laws and rescue archaeology; there are a large number of researchers working in this area and time slice.

*Coverage moderate*: characterizes grid squares where a few areas have been closely studied but large areas of spatial uncertainty remain. There may be ‘islands' of data amid a sea of unexplored land, but there are several such islands.

*Coverage poor*: Islands of data are fewer and smaller. Little to no archaeological survey has been conducted, or else visibility or taphonomic issues are severe.

Quality variables are:

*Quality high*: Most sites have been well-studied, with secure dating and analysis of floral and faunal remains. While uncertainties certainly exist, and some sites were studied prior to the development of modern methods, there is broad consensus about topics such as mode of subsistence and the use of specific domesticates. Chronological quality is high and often understood by reference to high numbers of radiocarbon determinations and/or other high-precision dating methods.

*Quality variable:* The distribution of sites is reasonably well-known, and many or most have been surveyed. Dating is based on artifact typologies and therefore chronological quality is moderate. This category should also be used where there is limited information about floral and faunal remains.

*Quality poor:* There are severe problems of chronology and/or plant and animal remains are rarely studied.

References Cited:

1. Harris DR, Fuller DQ (2014) Agriculture: definition and overview. In: Encyclopedia of global archaeology. Springer, New York, pp 104–113

2. Klein-Goldewijk K, Beusen A, Doelman J, Stehfest E (2017) Anthropogenic land use estimates for the Holocene - HYDE 3.2. Earth System Science Data 9:927–953

3. Myrdal J (2011) Farming and feudalism, 1000–1700. In: Myrdel J, Morell M (eds) The Agrarian History of Sweden: 4000 bc to ad 2000. Nordic Academic Press, Lund, pp 72–117

4. Richards P (1985) Indigenous agricultural Revolution: Ecology and food production in West Africa. Hutchinson, London

5. Bogaard A (2004) Neolithic farming in Central Europe: an archaeobotanical study of crop husbandry practices. Routledge, London

6. Midant-Reynes B (2000) The Prehistory of Egypt. Blackwell, Oxford

7. Bellwood PS (2005) Chapter 3: The Beginnings of Agriculture in Southwest Asia. First Farmers: The Origins of Agricultural Societies

8. Cruz P, Winkel T, Ledru M-P, Bernard C, Egan N, Swingedouw D, Joffre R (2017) Rain-fed agriculture thrived despite climate degradation in the pre-Hispanic arid Andes. Sci Adv 3:e1701740

9. Conklin H (1964) The Study of Shifting Cultivation. Union Panamericana, Washington D.C.

10. Nair PKR (1993) An introduction to agroforestry. Kluwer Academic Publishers, Dordrecht

11. Atangana A, Khasa D, Chang S, Degrande A (2014) Tropical Agroforestry. https://doi.org/10.1007/978-94-007-7723-1

12. Mertz O, Padoch C, Fox J, Cramb RA, Leisz SJ, Lam NT, Vien TD (2009) Swidden Change in Southeast Asia: Understanding Causes and Consequences. Hum Ecol 37:259–264

13. Nigh R, Diemont SA (2013) The Maya milpa: fire and the legacy of living soil. Frontiers in Ecology and the Environment. https://doi.org/10.1890/120344

14. Allan W (1965) The African Husbandman. Oliver and Boyd, Edinburgh

15. Kakeya M, Sugiyama Y (1985) Citemene, Figure Millet and Bemba Culture: A Socio- Ecological Study of Slash-and-Burn Cultivation in Northeastern Zambia. African Study Monographs Supplementary Issue 4:1–24

16. Thaler GM, Anandi CAM (2017) Shifting cultivation, contentious land change and forest governance: the politics of swidden in East Kalimantan. The Journal of Peasant Studies 44:1066–1087

17. Kurien AJ, Lele S, Nagendra H (2019) Farms or Forests? Understanding and Mapping Shifting Cultivation Using the Case Study of West Garo Hills, India. Land 8:133

18. Barnes GL (1990) Paddy Soils Now and Then. World Archaeology 22:1–17

19. Rostain S (2012) Islands in the rainforest: landscape management in pre-Columbian Amazonia. Left Coast Press, Inc, Walnut Creek, CA

20. Chen H, Zhu Q, Peng C, et al (2013) Methane emissions from rice paddies natural wetlands, lakes in China: synthesis new estimate. Glob Change Biol 19:19–32

21. Morrison KD (2015) Archaeologies of flow: Water and the landscapes of Southern India past, present, and future. Journal of Field Archaeology 40:560–580

22. Morrison KD (2009) Daroji Valley: Landscape, Place, and the Making of a Dryland Reservoir System. Manohar, Delhi

23. Bray F (1994) The rice economies: technology and development in Asian societies. University of California Press, Berkeley

24. Kirch PV (1996) Late Holocene human-induced modifications to a central Polynesian island ecosystem. Proceedings of the National Academy of Sciences 93:5296–5300

25. Bayliss-Smith TP, Hviding E (2015) Landesque capital as an alternative to food storage in Melanesia: Irrigated taro terraces in New Georgia, Solomon Islands. Environmental Archaeology 20:425–436

26. Kay AU, Fuller DQ, Neumann K, et al (2019) Diversification, Intensification and Specialization: Changing Land Use in Western Africa from 1800 BC to AD 1500. J World Prehist 32:179–228

27. Carney JA (2001) African Rice in the Columbian Exchange. J Afr Hist 42:377–396

28. Catling D (1993) Rice in Deep Water. Palgrave Macmillan Limited, London

29. Magnavita C (2002) Recent archaeological finds of domesticated Sorghum bicolor in the Lake Chad region. Nyame Akuma 57:14–20

30. Gronenborn D (2001) Masakwa in the Chad Basin - An examination of the archaeological and historical sources. In: Kahlber S, Neumann K (eds) Man and environment in the West African Sahel. Berichte des Sonderforschungsbereichs, Frankfurt am Main, pp 73–84

31. Denevan WM (1970) Aboriginal Drained-Field Cultivation in the Americas: Pre-Columbian reclamation of wet lands was widespread in the savannas and highlands of Latin America. Science 169:647–654

32. Robles B, Flores J, Martínez JL, Herrera P (2018) The Chinampa: An Ancient Mexican Sub‐Irrigation System. Irrig and Drain 115–122

33. Thesiger W (1964) The Marsh Arabs. E.P. Dutton, New York

34. McIntosh RJ (1998) Peoples of the Middle Niger. Blackwell, Oxford

35. Leakey R (1996) Definition of agroforestry revisited. Agroforestry Today 8:5–7

36. Gliessman SR (2007) Agroecology: the ecology of sustainable food systems, 2nd ed. CRC Press, Boca Raton

37. Terrell JE, Hart JP, Barut S, et al (2003) Domesticated Landscapes: The Subsistence Ecology of Plant and Animal Domestication. Journal of Archaeological Method and Theory 10:323–368

38. Kaniewski D, Van Campo E, Boiy T, Terral J-F, Khadari B, Besnard G (2012) Primary domestication and early uses of the emblematic olive tree: palaeobotanical, historical and molecular evidence from the Middle East. Biological Reviews 87:885–899

39. Tengberg M (2012) Beginnings and early history of date palm garden cultivation in the Middle East. Journal of Arid Environments 86:139–147

40. Logan AL, D’Andrea AC (2012) Oil palm, arboriculture, and changing subsistence practices during Kintampo times (3600–3200 BP, Ghana). Quaternary International 249:63–71

41. Hunt CO, Rabett RJ (2014) Holocene landscape intervention and plant food production strategies in island and mainland Southeast Asia. Journal of Archaeological Science 51:22–33

42. Gourou P (1949) Observações geográficas na Amazônia. Revista Brasileira de Geografia 11:354–408

43. Fraser JA, Leach M, Fairhead J (2014) Anthropogenic Dark Earths in the Landscapes of Upper Guinea, West Africa: Intentional or Inevitable? Annals of the Association of American Geographers 104:1222–1238

44. Frausin V, Fraser JA, Narmah W, Lahai MK, Winnebah TRA, Fairhead J, Leach M (2014) “God Made the Soil, but We Made It Fertile”: Gender, Knowledge, and Practice in the Formation and Use of African Dark Earths in Liberia and Sierra Leone. Hum Ecol 42:695–710

45. Neves EG, Petersen JB, Bartone RN, Da Silva CA (2003) Historical and socio-cultural origins of Amazonian Dark Earths. In: Lehman J, Kern DC, Glaser B, Woods WI (eds) Amazonian Dark Earths: Origin, Properties, Management. Springer, Dordrecht, pp 29–50

46. Söderström M, Eriksson J, Isendahl C, Schaan DP, Stenborg P, Rebellato L, Piikki K (2016) Sensor mapping of Amazonian Dark Earths in deforested croplands. Geoderma 281:58–68

47. Clement CR, Denevan WM, Heckenberger MJ, Junqueira AB, Neves EG, Teixeira WG, Woods WI (2015) The domestication of Amazonia before European conquest. Proc R Soc B 282:20150813

48. Erickson CL (2008) Amazonia: The Historical Ecology of a Domesticated Landscape. In: Silverman H, Isbell WH (eds) The Handbook of South American Archaeology. Springer, New York, pp 157–183

49. Glaser B, Birk JJ (2012) State of the scientific knowledge on properties and genesis of Anthropogenic Dark Earths in Central Amazonia (terra preta de Índio). Geochimica et Cosmochimica Acta 82:39–51

50. Hemp A (2006) The Banana Forests of Kilimanjaro: Biodiversity and Conservation of the Chagga Homegardens. Biodivers Conserv 15:1193–1217

51. Pinho RC, Alfaia SS, Miller RP, Uguen K, Magalhães LD, Ayres M, Freitas V, Trancoso R (2011) Islands of fertility: Soil improvement under indigenous homegardens in the savannas of Roraima, Brazil. Agroforest Syst 81:235–247

52. Maxwell JJ, Howarth JD, Vandergoes MJ, Jacobsen GE, Barber IG (2016) The timing and importance of arboriculture and agroforestry in a temperate East Polynesia Society, the Moriori, Rekohu (Chatham Island). Quaternary Science Reviews 149:306–325

53. Whitehouse NJ, Smith D (2010) How fragmented was the British Holocene wildwood? Perspectives on the “Vera” grazing debate from the fossil beetle record. Quaternary Science Reviews 29:539–553

54. Smith D, Nayyar K, Schreve D, Thomas R, Whitehouse N (2014) Can dung beetles from the palaeoecological and archaeological record indicate herd concentration and the identity of herbivores? Quaternary International 341:119–130

55. Anderson RS, Ejarque A, Rice J, Smith SJ, Lebow CG (2015) Historic and Holocene Environmental Change in the San Antonio Creek Basin, Mid-coastal California. Quat res 83:273–286

56. Ejarque A, Anderson RS, Simms AR, Gentry BJ (2015) Prehistoric fires and the shaping of colonial transported landscapes in southern California: A paleoenvironmental study at Dune Pond, Santa Barbara County. Quaternary Science Reviews 112:181–196

57. Shahack-Gross R (2011) Herbivorous livestock dung: formation, taphonomy, methods for identification, and archaeological significance. Journal of Archaeological Science 38:205–218

58. Bird-David N (2015) Hunting and Gathering Societies: Anthropology. In: International Encyclopedia of the Social & Behavioral Sciences. Elsevier, pp 428–431

59. Morrison KD (2002) General Introduction: Historicizing Adaptation, Adapting to History. Forager-Traders in South and Southeast Asia: Long-Term Histories

60. Lee G-A (2011) The Transition from Foraging to Farming in Prehistoric Korea. Current Anthropology 52:S307–S329

61. Kent S (ed) (2008) Farmers as Hunters: theThe Implications of Sedentism. Cambridge University Press, Cambridge

62. Out WA (2008) Growing habits? Delayed introduction of crop cultivation at marginal Neolithic wetland sites. Veget Hist Archaeobot 17:131–138

63. Antolín F, Bleicher N, Brombacher C, Kühn M, Steiner BL, Jacomet S (2016) Quantitative approximation to large-seeded wild fruit use in a late Neolithic lake dwelling: New results from the case study of layer 13 of Parkhaus Opéra in Zürich (Central Switzerland). Quaternary International 404:56–68

64. Antolín F, Jacomet S (2015) Wild fruit use among early farmers in the Neolithic (5400–2300 cal bc) in the north-east of the Iberian Peninsula: an intensive practice? Veget Hist Archaeobot 24:19–33

65. Marlowe F (2010) The Hadza: hunter-gatherers of Tanzania. University of California Press, Berkeley

66. Smith BD (2001) Low-Level Food Production. Journal of Archaeological Research 9:1–43

67. Bettinger RL (1977) Aboriginal Human Ecology in Owens Valley: Prehistoric Change in the Great Basin. Am antiq 42:3–17

68. Price TD, Brown JA (eds) (1985) Prehistoric hunter-gatherers: the emergence of cultural complexity. Academic Press, Orlando

69. Blackburn TC, Anderson K (eds) (1993) Before the wilderness: environmental management by native Californians. Ballena Press, Menlo Park

70. Willcox G, Stordeur D (2012) Large-scale cereal processing before domestication during the tenth millennium cal BC in northern Syria. Antiquity 86:99–114

71. Price TD (2000) The introduction of farming in North Europe. Europe’s First Farmers

72. Holl A (2005) Holocene “aquatic” adaptations in tropical North Africa. In: Stahl EB (ed) African Archaeology: A Critical introduction. Blackwell, Oxford, pp 174–86

73. Sutton JEG (1974) The Aquatic Civilization of Middle Africa. J Afr Hist 15:527–546

74. Dale D, Ashley CZ (2010) Holocene hunter-fisher-gatherer communities: new perspectives on Kansyore Using communities of Western Kenya. Azania: Archaeological Research in Africa 45:24–48

75. Prendergast ME (2010) Kansyore fisher-foragers and transitions to food production in East Africa: the view from Wadh Lang’o, Nyanza Province, Western Kenya. Azania: Archaeological Research in Africa 45:83–111

76. Robertshaw P, Collett D, Gifford D, Mbae NB (1983) Shell Middens on the Shores of Lake Victoria. Azania: Archaeological Research in Africa 18:1–43

77. Rowley-Conwy P, Layton R (2011) Foraging and farming as niche construction: stable and unstable adaptations. Phil Trans R Soc B 366:849–862

78. Smith BD (2011) The Cultural Context of Plant Domestication in Eastern North America. Current Anthropology 52:S471–S484

79. Crawford GW (2008) The Jomon in early agriculture discourse: issues arising from Matsui, Kanehara and Pearson. World Archaeology 40:445–465

80. Louwe Kooijmans LP (2007) The gradual transition to farming in the Lower Rhine Basin. Proceedings of the British Academy 144:287–309

81. Dusseldorp GL, Amkreutz LWSW A long slow goodbye – Re-examining the Mesolithic – Neolithic transition (5500 – 2500 BCE) in the Dutch delta. In: Klinkenberg MV, van Oosten RMR, van Driel-Murray C (eds) A Human Environment. Studies in honour of 20 years Analecta editorship by prof. dr. Corrie Bakels. Sidestone Press, Leiden, pp 121–142

82. Out WA (2009) Sowing the seed? human impact and plant subsistence in Dutch wetlands during the Late Mesolithic and Early and Middle Neolithic (5500 - 3400 cal BC). University Press, Leiden

83. Bakels C, Zeiler J The fruits of the land: Neolithic subsistence. In: Louwe Kooijmans LP, Van den Broeke PW, Fokkens H, Van Gijn AL (eds) The prehistory of the Netherlands. Amsterdam University Press, Amsterdam, pp 311–336

84. Malindine J (2019) Prehistoric Aquaculture: Origins, Implications, and an Argument for Inclusion. Cult Agric Food Environ 41:66–70

85. Nash CE (2011) The history of aquaculture. Wiley-Blackwell, Ames

86. Burney DA (2002) Late Quaternary Chronology and Stratigraphy of Twelve Sites On Kaua‘i. Radiocarbon 44:13–44

87. Simmons IG, Innes JB (1996) Disturbance Phases in the Mid-Holocene Vegetation at North Gill, North York Moors: Form and Process. Journal of Archaeological Science 23:183–191

88. Wilkinson TJ, Bintliff J, Curvers HH, et al (1994) The Structure and Dynamics of Dry-Farming States in Upper Mesopotamia [and Comments and Reply]. Current Anthropology 35:483–520

89. Browman DL (1989) Origins and development of Andean pastoralism: an overview of the last 6000 years. In: Clutton-Brock J (ed). Unwin Hyman, London, pp 256–268

90. Franklin WL (1982) Biology, ecology, and relationship to man of the South American camelids. In: Mares MA, Genoways HH (eds) Mammalian biology in South America. University of Pittsburgh., Pittsburg, pp 457–489

91. Evans SM, Carter S, Yeo B, Glenbow Museum (eds) (2000) Cowboys, ranchers, and the cattle business: cross-border perspectives on ranching history. University of Calgary Press, Calgary

92. Sluyter A (2015) How Africans and Their Descendants Participated in Establishing Open-Range Cattle Ranching in the Americas. environ hist camb 21:77–101

93. Paterson A (2018) Once were foragers: The archaeology of agrarian Australia and the fate of Aboriginal land management. Quaternary International 489:4–16

94. Mills PR, White CL, Barna B (2013) The Paradox of the Paniolo: An Archaeological Perspective of Hawaiian Ranching. Hist Arch 47:110–132

95. Gadgil M, Malhotra KC (1982) Ecology of a pastoral caste: Gavli Dhangars of peninsular India. Hum Ecol 10:107–143

96. Brasseur G (1968) Les établissements humains au Mali. Institut Fondementale d’Afrique Noire, Dakar

97. Costello E, Svensson E (eds) (2018) Historical Archaeologies of Transhumance across Europe, 1st ed. https://doi.org/10.4324/9781351213394

98. Carrer F (2015) Herding Strategies, Dairy Economy and Seasonal Sites in the Southern Alps: Ethnoarchaeological Inferences and Archaeological Implications. jmea 28:3–22

99. Wehling K (1992) Vorder Orient: Nomadismus und andere Formen der Wanderviehwirtschaft (Middle East: Nomadism and other Forms of Pastoral Migration).

100. Riemer H (2010) Archäologischer Survey auf dem südlichen Abu-Muharik-Plateau. In: Kindermann K (ed) Djara. Zur mittelholozänen Besiedlungsgeschichte zwischen Niltal und Oasen (Abu-Muharik-Plateau, Ägypten). Heinrich-Barth-Institut, Köln, pp 547–713

101. Gallinaro M (2018) Mobility and pastoralism in the Egyptian Western Desert: Steinplätze in the Holocene regional settlement patterns. All’insegna del giglio, Sesto Fiorentino (FI)

102. Di Lernia S (1999) Discussing pastoralism. The case of the Acacus and surroundings (Libyan Sahara). Sahara 11:7–20

103. Biagetti S, di Lernia S (2003) Vers un modèle ethnographique-écologique d’une société pastorale préhistorique Saharienne. Sahara 14:7–30

104. Adriansen HK, Nielsen TT (2002) Going Where the Grass Is Greener: On the Study of Pastoral Mobility in Ferlo, Senegal. Human Ecology 30:215–226

105. Ingold T (1980) Hunters, Pastoralists and Ranchers: Reindeer Economies and their Transformations, 1st ed. https://doi.org/10.1017/CBO9780511558047

106. Hermes TR, Frachetti MD, Doumani Dupuy PN, Mar’yashev A, Nebel A, Makarewicz CA (2019) Early integration of pastoralism and millet cultivation in Bronze Age Eurasia. Proc R Soc B 286:20191273

107. Dupire M (1962) Peuls nomades: étude descriptive des Wodaabe du Sahel Nigérien. Institut d’Ethnologie, Paris

108. Homewood KM, Rogers WA (1991) Maasailand ecology: Pastoralist development and wildlife conservation in Ngorongoro, Tanzania. Cambridge University Press, Cambridge

109. Mccarthy N, Di Gregorio M (2007) Climate variability and flexibility in resource access: the case of pastoral mobility in Northern Kenya. Envir Dev Econ 12:403–421

110. Stenning DJ (1959) Savannah Nomads. International African Institute, London

111. Biagetti S (2014) Ethnoarchaeology of the Kel Tadrart Tuareg Pastoralism and Resilience in Central Sahara. Springer, Heidelberg

112. Cole DP (1975) Nomads of the Nomads: the Al Murrah Bedouin of the empty quarter. AHM Publishing, Arlington Heights

113. Gordon RB, Malone PM (1997) Texture of industry: an archaeological view of industrialization of North America. Oxford University Press, Oxford

114. Scarborough VL (2003) Flow of power: ancient water systems and landscapes. SAR Press, Santa Fe

115. Fletcher R, Penny D, Evans D, Pottier C, Barbetti M, Kummu M, Lustig T, Authority for the Protection and Management of Angkor and the Region of Siem Reap (APSARA) Department of Monuments and Archaeology Team (2008) The water management network of Angkor, Cambodia. Antiquity 82:658–670

116. Horton M, Middleton J (2000) The Swahili: the social landscape of a mercantile society. Blackwell Publishers, Oxford, UK ; Malden, Mass

117. Kay AU, Kaplan JO (2015) Human subsistence and land use in sub-Saharan Africa, 1000BC to AD1500: A review, quantification, and classification. Anthropocene 9:14–32

118. Keene D (2012) Medieval London and its supply hinterlands. Reg Environ Change 12:263–281

119. Rojas JL de (2012) Tenochtitlan: capital of the Aztec empire. University Press of Florida, Gainseville

120. Coulston JC, Dodge H (eds) (2000) Ancient Rome: the archaeology of the eternal city. Oxford University School of Archaeology, Oxford

121. Cooke CA, Abbott MB, Wolfe AP (2008) Late-Holocene atmospheric lead deposition in the Peruvian and Bolivian Andes. The Holocene 18:353–359

122. Cooke CA, Balcom PH, Kerfoot C, Abbott MB, Wolfe AP (2011) Pre-Colombian Mercury Pollution Associated with the Smelting of Argentiferous Ores in the Bolivian Andes. AMBIO 40:18–25

123. Robins NA (2011) Mercury, mining, and empire: the human and ecological cost of colonial silver mining in the Andes. Indiana University Press, Bloomington

124. Vicent García JM, Martínez Navarrete M^a^ I, López Sáez JA, De Zavala Morencos I (2010) Impacto medioambiental de la minería y la metalurgia del cobre durante la Edad del Bronce en Kargaly (región de Orenburgo, Rusia). Trab prehist 67:511–544

125. Childs ST, Herbert E (2005) Metallurgy and its consequences. In: Stahl AB (ed) African Archaeology: A Critical Introduction. Blackwell, Oxford, pp 276–300

126. Flad RK (2008) Rethinking the Context of Production through an Archaeological Study of Ancient Salt Production in the Sichuan Basin, China. Archeological Papers of the American Anthropological Association 17:108–128

127. Dangal SRS, Tian H, Zhang B, Pan S, Lu C, Yang J (2017) Methane emission from global livestock sector during 1890-2014: Magnitude, trends and spatiotemporal patterns. Glob Change Biol 23:4147–4161

128. Lawrence PJ, Feddema JJ, Bonan GB, et al (2012) Simulating the Biogeochemical and Biogeophysical Impacts of Transient Land Cover Change and Wood Harvest in the Community Climate System Model (CCSM4) from 1850 to 2100. J Climate 25:3071–3095

129. Dufraisse A (2008) Firewood management and woodland exploitation during the late Neolithic at Lac de Chalain (Jura, France). Veget Hist Archaeobot 17:199–210

130. Iles L (2016) The Role of Metallurgy in Transforming Global Forests. J Archaeol Method Theory 23:1219–1241

131. Van Oost K, Govers G, Quine TA, Heckrath G, Olesen JE, De Gryze S, Merckx R (2005) Landscape-scale modeling of carbon cycling under the impact of soil redistribution: The role of tillage erosion. Global Biogeochem Cycles 19:GB4014

132. Sanderman J, Hengl T, Fiske GJ (2017) Soil carbon debt of 12,000 years of human land use. Proc Natl Acad Sci USA 114:9575–9580

133. Rommens T, Verstraeten G, Lang A, Poesen J, Govers G, Van Rompaey A, Lang A, Peeters I (2005) Soil erosion and sediment deposition in the Belgian oess belt during the Holocene: establishing a sediment budget for a small agricultural catchment. The Holocene 15:1032–1043

134. Price TD (2015) Ancient Scandinavia: an archaeological history from the first humans to the Vikings. Oxford University Press, Oxford

135. Potts DT (1994) Contributions to the agrarian history of Eastern Arabia I. Implements and cultivation techniques. Arab Arch Epigraphy 5:158–168

136. López AG (2010) New evidence on the use of implements in al-Madām area, Sharjah, UAE. Proceedings of the Seminar for Arabian Studies 40:171–174

137. López-Bultó O, Piqué R, Antolín F, Barceló JA, Palomo A, Clemente I (2020) Digging sticks and agriculture development at the ancient Neolithic site of la Draga (Banyoles, Spain). Journal of Archaeological Science: Reports 30:102193

138. Bogaard A, Hodgson JG, Wilson PJ, Band SR (1998) An index of weed size for assessing the soil productivity of ancient crop fields. Veget Hist Archaebot 7:17–22

139. Bogaard A, Palmer C, Jones G, Charles M, Hodgson JG (1999) A FIBS Approach to the Use of Weed Ecology for the Archaeobotanical Recognition of Crop Rotation Regimes. Journal of Archaeological Science 26:1211–1224

140. Charles M, Jones G, Hodgson JG (1997) FIBS in Archaeobotany: Functional Interpretation of Weed Floras in Relation to Husbandry Practices. Journal of Archaeological Science 24:1151–1161

141. Jones G, Charles M, Bogaard A, Hodgson J (2010) Crops and weeds: the role of weed functional ecology in the identification of crop husbandry methods. Journal of Archaeological Science 37:70–77

142. Fuller DQ, Stevens CJ, McClatchie M (2014) Routine Activities, Tertiary Refuse and Labour Organisation: social inferences from everyday archaeobotany. In: Madella M, Lancelotti C, Savard M (eds) Ancient Plants and People, Contemporary Trends in Archaeology. University of Arizona Press, Tuscon, pp 174–217.

143. Deák J, Gebhardt A, Lewis H, Usai MR, Lee H (2017) Soils Disturbed by Vegetation Clearance and Tillage. In: Nicosia C, Stoops G (eds) Archaeological Soil and Sediment Micromorphology. John Wiley & Sons, Ltd, Chichester, UK, pp 231–264

144. van Andel TH, Zangger E, Demitrack A (1990) Land Use and Soil Erosion in Prehistoric and Historical Greece. Journal of Field Archaeology 17:379

145. Borejsza A (2013) Village and field abandonment in post-Conquest Tlaxcala: A geoarchaeological perspective. Anthropocene 3:9–23

146. Ayala G, French C (2005) Erosion modeling of past land-use practices in the Fiume di Sotto di Troina river valley, north-central Sicily. Geoarchaeology 20:149–167

147. Heckmann M (2014) Farmers, smelters and caravans: Two thousand years of land use and soil erosion in North Pare, NE Tanzania. CATENA 113:187–201

148. French C, Sulas F, Madella M (2009) New geoarchaeological investigations of the valley systems in the Aksum area of northern Ethiopia. CATENA 78:218–233

149. Venclová N, Dreslerová D (2013) Iron production, settlement, and environment: a regional approach. In: Rausz S, Colin A, Gruel K, Ralston I, Dechezleprê T (eds) L’âge du Fer en Europe. Ausonius Éditions Mémoires, Bordeaux, pp 291–304

150. Jouffroy-Bapicot I, Pulido M, Baron S, Galop D, Monna F, Lavoie M, Ploquin A, Petit C, de Beaulieu J-L, Richard H (2007) Environmental impact of early palaeometallurgy: pollen and geochemical analysis. Veget Hist Archaeobot 16:251–258

151. Sivramkrishna S (2009) Production Cycles and Decline in Traditional Iron Smelting in the Maidan, Southern India, c. 1750-1950: An Environmental History Perspective. Environment and History 15:163–197

152. Marston JM (2009) Modeling wood acquisition strategies from archaeological charcoal remains. Journal of Archaeological Science 36:2192–2200

153. Eichhorn B, Robion-Brunner C (2017) Wood exploitation in a major pre-colonial West African iron production centre (Bassar, Togo). Quaternary International 458:158–177

154. Iles L (2020) Exploring the impact of iron production on forest and woodland resources: estimating fuel consumption from slag. STAR: Science & Technology of Archaeological Research 1–21

155. Pyne SJ (1997) World fire: the culture of fire on earth. University of Washington Press, Seattle

156. Bliege Bird R, Bird DW, Codding BF, Parker CH, Jones JH (2008) The “fire stick farming” hypothesis: Australian Aboriginal foraging strategies, biodiversity, and anthropogenic fire mosaics. Proceedings of the National Academy of Sciences 105:14796–14801

157. Roos CI, Zedeño MN, Hollenback KL, Erlick MMH (2018) Indigenous impacts on North American Great Plains fire regimes of the past millennium. Proc Natl Acad Sci USA 115:8143–8148

158. Power MJ, Marlon JR, Bartlein PJ, Harrison SP (2010) Fire history and the Global Charcoal Database: A new tool for hypothesis testing and data exploration. Palaeogeography, Palaeoclimatology, Palaeoecology 291:52–59

159. Marlon JR, Kelly R, Daniau A-L, et al (2016) Reconstructions of biomass burning from sediment-charcoal records to improve data–model comparisons. Biogeosciences 13:3225–3244

160. Blarquez O, Vannière B, Marlon JR, Daniau A-L, Power MJ, Brewer S, Bartlein PJ (2014) paleofire: An R package to analyse sedimentary charcoal records from the Global Charcoal Database to reconstruct past biomass burning. Computers & Geosciences 72:255–261

161. Marlon JR, Bartlein PJ, Daniau A-L, Harrison SP, Maezumi SY, Power MJ, Tinner W, Vanniére B (2013) Global biomass burning: a synthesis and review of Holocene paleofire records and their controls. Quaternary Science Reviews 65:5–25

162. Arnold D (2020) Fire, Forest, City: A Social Ecology of Fire in British India. environ hist camb. https://doi.org/10.3197/096734019X15463432086991

163. Cuthrell RQ, Striplen C, Hylkema M, Lightfoot KG (2016) A land of fire: Anthropogenic burning on the central coast of California.

164. Lightfoot KG, Cuthrell RQ (2015) Anthropogenic burning and the Anthropocene in late-Holocene California. The Holocene 25:1581–1587

165. Sluyter A, Duvall C (2016) African Fire Cultures, Cattle Ranching, and Colonial Landscape Transformations in the Neotropics. Geographical Review 106:294–311

166. Hunter R, Sluyter A (2015) Sixteenth-century soil carbon sequestration rates based on Mexican land-grant documents. The Holocene 25:880–885

167. Sluyter A (2012) Black Ranching Frontiers: African Cattle Herders of the Atlantic World, 1500–1900. Yale University Press, New Haven

168. Webley L, Vander Linden M, Haselgrove C, Bradley R (eds) (2012) Development-led archaeology in northwest Europe: proceedings of a round table at the University of Leicester, 19th-21st November 2009. Oxbow Books, Oxford

169. Jacomet S, Ebersbach R, Akeret Ö, et al (2016) On-site data cast doubts on the hypothesis of shifting cultivation in the late Neolithic (c. 4300–2400 cal. BC): Landscape management as an alternative paradigm. The Holocene 26:1858–1874

**Supplemental Material S2**

**LandUse6K Database Documentation**

**Introduction**

The generation of digital global maps of ancient and historical land-use practices for designated time periods is the central goal of the land-use group within LandCover6k. To facilitate the creation of these maps, we prepared a geodatabase in ESRI’s ArcGIS that allows LandCover6k (LC6k) participants and regional working groups to record data on historical and ancient land-use in a standardized way, using identical terminology, spatial units, and data formats. The database operationalizes the land-use classification system and land-use mapping principles detailed in the main text of the paper. We provide basic documentation of the database here.

**Spatial Units and Data Format**

The database is built around global 8 x 8 km grids of squares. One global grid of these squares exists for each of the time slices considered by LC6k. Land use classifications, variables, and data quality assessments are recorded for individual squares and are considered to be a general assessment for the land that falls within that square at the designated time period.

The global grids have been created in ArcGIS as polygon geodatabase feature classes, to allow for complex attribute tables and data validation procedures. The project uses the Eckert IV projection system, chosen because it preserves relative area measurements. Following completion of land-use mapping, these vector files will be converted to raster datasets for integration with comparable file formats generated by the pollen-based land-cover reconstructions and with output produced by the climate and ALCC modeling communities.

This grid-based approach was designed to ensure group members would record assessments at consistent scales, provide the capacity for global coverage, and enable quick map production. At the beginning of the LC6k project, many archaeologists and historians expressed a desire to “draw what [they] know” using freeform polygons. However, this “blank canvas,” approach resulted in three difficulties. First, different individuals and groups, given similar background information and instructions, produced significantly different outputs, particularly in regard to spatial resolution. Second, the variable quality of data in different regions resulted in some groups spending a lot of time representing the complexities of small, well-studied areas in complex polygons while spending minimal time considering larger, less well-studied areas. While the resolution and quality of the historical and ancient data available for different regions are indeed highly variable, LC6k aims to create global maps, and group members must devote time to providing assessments for all areas without becoming overly involved in the minutiae of the well-known. Third, data entry proceeded slowly. The 8 x 8 km squares allow participants to produce uniform datasets more quickly, in a format that is easier to compare with data from other researchers. An additional advantage to this approach is that it does not require participants to share their own databases and archaeological data points. Rather than asking participants to replicate the high-resolution details contained in their own unpublished or not-fully-published datasets in irregular polygon form, we ask them to summarize what they know to create a more synthetic but less detailed map formed from the 8 x 8 km squares.

The size of the squares—8 x 8 km—is a compromise between the needs of the ALCC modeling, pollen, and archaeological communities involved in the LC6K project. As archaeological data are frequently highly spatially heterogeneous, and archaeologists often request the smallest grid size possible, we are recording data at the smallest scale used within the larger LC6k community. The pollen community within LC6K, for example, is producing land-cover rasters with a resolution of 1 degree. The smallest grids used by the ALCC modelers are 5 minutes (1/12 of a degree) so we chose a grid that closely matches this (see Fig 8).

**Classifications and Variables**

The classification system described in the body of the paper has been set in the database in the form of drop-down menus (in the terminology of ESRI’s ArcGIS, these are called “domains”) containing all of the valid options for each level of the land use classification hierarchy (LU1, LU2, LU3) and all of the valid options for each of the seven variables relevant across land-use systems. The database additionally contains two fields for recording data quality and data coverage. Again, these classifications and variables are recorded by individual 8 x 8 km squares. Various selection and calculation tools in ArcGIS make it possible to simultaneously classify and edit the variables for a group of grid squares that should be given the same values.

The menus are contextual at the LU1 level, meaning that once the LU1 classification has been selected, the options for the LU2 and variable menus are restricted to be those that fall under that LU1 category (in the terminology of ESRI’s ArcGIS, the LU1 defines a “subtype”). For example, if one selects “Agriculture” as the LU1 category, only LU2 agriculture variables (i.e., “herbaceous/ground crops,” “swidden/shifting,” “agroforestry/arboriculture,” “wet cultivation”) will be options in the LU2 menu and the LU2 options for other LU1 categories like Pastoralism and Hunting-Gathering-Fishing-Foraging will not be options.

**Descriptions of Coded Values**

Within the geodatabase, land use classifications and variables are recorded in the feature class attribute table using coded values. The tables below provide the values for those codes.

Regional Working Group

| Code | Description |
| --- | --- |
| 0 | no selection |
| 1 | North America |
| 2 | Central America |
| 3 | Amazonia |
| 4 | South America outside Amazonia |
| 5 | Africa |
| 6 | Europe |
| 7 | West Asia/Middle East |
| 8 | Central Asia |
| 9 | North and East Asia |
| 10 | South and Southeast Asia |
| 11 | Australia and Oceania |

LC6k Time Slice

| Code | Description |
| --- | --- |
| 0 | no selection |
| 1 | 6000 BP |
| 2 | 3000 BP |
| 3 | 1000 BP |
| 4 | 1500 CE |
| 5 | 1850 CE |

No Human Land Use

| Code | Description |
| --- | --- |
| 0 | no selection |
| 1 | underwater |

Agriculture LU2

| Code | Description |
| --- | --- |
| 0 | no selection |
| 1 | agroforestry/arboriculture |
| 2 | swidden/shifting |
| 3 | herbaceous/ground crops |
| 4 | wet cultivation |

Hunting-Gathering-Fishing-Foraging LU2

| Code | Description |
| --- | --- |
| 0 | no selection |
| 1 | HGF only |
| 2 | broad-based and aquatic resources |
| 3 | low-level food production |
| 4 | specialized fish production |

Pastoralism LU2

| Code | Description |
| --- | --- |
| 0 | no selection |
| 1 | anchored |
| 2 | ranching |
| 3 | mobile regular |
| 4 | mobile irregular |

Urban/Extractive Industries LU2

| Code | Description |
| --- | --- |
| 0 | no selection |
| 1 | dispersed urban/peri-urban |
| 2 | dense urban |
| 3 | mining/quarry |

Cultigens Variable

| Code | Description |
| --- | --- |
| 0 | no selection |
| 1 | wheat/barley/pulses |
| 2 | millets/pulses |
| 3 | maize/beans/squash |
| 4 | Mediterranean complex olives/vine/wheat |
| 5 | vines |
| 6 | rice (dry/hill) |
| 7 | rice (wet/paddy) |
| 8 | tubers |
| 9 | millets/teff/other similar |
| 10 | maize |
| 11 | orchard/tree crops/dates |
| 12 | sugar cane |
| 13 | oats |
| 14 | rye |
| 15 | sago |
| 16 | cotton |
| 17 | other |

Animals Variable

| Code | Description |
| --- | --- |
| 0 | no selection |
| 1 | goat and/or sheep |
| 2 | cattle |
| 3 | equids |
| 4 | camel |
| 5 | water buffalo |
| 6 | pigs |
| 7 | camelids, llama and/or alpaca |
| 8 | reindeer |
| 9 | other |

Water Modification Variable

| Code | Description |
| --- | --- |
| 0 | no selection |
| 1 | rainfed |
| 2 | flood |
| 3 | canals/channels |
| 4 | terracing |
| 5 | cistern/reservoir storage |
| 6 | wells |
| 7 | dams |
| 8 | qanats |
| 9 | drains/water reduction |
| 10 | other |

Tillage Variable

| Code | Description |
| --- | --- |
| 0 | no selection |
| 1 | digging stick/dibble |
| 2 | plow/ard |
| 3 | tractor |
| 4 | manuring |

Pyrotechnology Variable

| Code | Description |
| --- | --- |
| 0 | no selection |
| 1 | metal (copper/bronze/tin) |
| 2 | metal (iron/steel) |
| 3 | charcoal |
| 4 | faience/large-scale ceramic firing |

Settlement Mode Variable

| Code | Description |
| --- | --- |
| 0 | no selection |
| 1 | dispersed |
| 2 | aggregated |
| 3 | non-sedentary |
| 4 | urban centers present |

Fire Variable

| Code | Description |
| --- | --- |
| 0 | no selection |
| 1 | present--landscape scale burning |
| 2 | absent--landscape scale burning |
| 3 | debated--landscape scale burning |

Data Coverage Variable

| Code | Description |
| --- | --- |
| 0 | no selection |
| 1 | High |
| 2 | Moderate |
| 3 | Poor |

Data Quality Variable

| Code | Description |
| --- | --- |
| 0 | no selection |
| 1 | High |
| 3 | Poor |
| 2 | Variable |

**Data Availability**

Middle East land use data for 6 kya have been exported as a shapefile and have been made available through the PANGAEA Data Publisher for Earth & Environmental Science [https://doi.pangaea.de/10.1594/PANGAEA.922243]. A blank copy of the geodatabase (with the global grid and the classification system entered into the subtypes and domains) is provided here as a supplementary file.
